# Supplementary material for: Single-breath-hold 3D abdominal metabolic MRI enables label-free diagnosis of liver cancer
Source: Nat Commun. 2026 Mar 31;17:4661. doi: 10.1038/s41467-026-71124-5 (PMC13201843; doi:10.1038/s41467-026-71124-5)
Supplement: Supplementary file 1 — Supplementary Information [file 41467_2026_71124_MOESM1_ESM.pdf]

Supplementary methods

Quantification methods

1. SSE-CEST reconstruction theory and calculation of the spatial-spectral resolution
2. Introduction of single-pool LD quantification
3. Introduction of R1pho background quantification

Supplementary table

Supplementary Table 1.1. In vivo abdominal SSE-CEST protocols.

Supplementary Table 1.2. Quantitative Bland-Altman results in ex vivo porcine liver in Fig. 2.

Supplementary Table 1.3. Quantitative results of correlation coefficients in healthy volunteers in Fig. 4b.

Supplementary Table 1.4. Quantitative results of normalized mean squared error in healthy volunteers in Fig. 4c.

Supplementary figures

Part 1: Reconstruction basis of SSE-CEST framework and its compatibility

Supplementary Figure 1.1-1.3. Display of spectral basis and error comparison among adjacent slices.

Supplementary Figure 1.4. Flexibility of SSE-CEST with respect to readout types validated on ex-vivo porcine liver.

Supplementary Figure 1.5. Optimization of readout flip-angles for human subjects.

Supplementary Figure 1.6. Combination of SSE-CEST with 'keyhole' technique.

Supplementary Figure 1.7. Numerical simulation of CEST signal evolution for fast- and slow-exchange metabolites under SSE-CEST conditions.

Part 2: Ex-vivo porcine liver experiments

Supplementary Figure 2.1. Consistency of three z-ω trajectories on porcine liver.

Supplementary Figure 2.2-2.3. Comparison of reconstruction images among slices.

Part 3: Glycogen phantom experiments

Supplementary Figure 3.1. the glycogen phantom using saturation B1 of 2.0 μT.

Supplementary Figure 3.2. Slice-by-slice comparison ST (1.2 ppm) images in glycogen phantoms acquired using SSE-CEST and using conventional CEST.

Supplementary Figure 3.3. Slice-by-slice comparison ST (-1.2 ppm) images in glycogen phantoms acquired using SSE-CEST and using conventional CEST.

Supplementary Figure 3.4. Comparison of SSE-CEST Z-spectra among 3 slices in main Fig. 3d.

Supplementary Figure 3.5. glycogen phantom using the human acquisition protocol.

Part 4: Healthy volunteers

Supplementary Figure 4.1. Dixon-type 3D gradient-echo acquisition sequence for human abdominal SSE-CEST.

Supplementary Figure 4.2. the repeatability of SSE-CEST on 10 healthy human subjects.

Supplementary Figure 4.3. the slice-variation of SSE-CEST induced by short shot interval.
Supplementary Figure 4.4-4.5. the compatibility of human-liver SSE-CEST with other designated z-ω trajectories.

Part 5: Fasting experiments

Supplementary Figure 5.1. The trajectories of Zneg (0.7 μT) and Zpos (0.7 μT) for fasting experiments and patients.
Supplementary Figure 5.2-5.4. LD quantification details on fasting experiments (0.7 μT).
Supplementary Figure 5.5. Rex quantification on fasting experiments (0.7 μT).
Supplementary Figure 5.6. Comparison of 0.7 μT and 2 μT.
Supplementary Figure 5.7. Multi-organ metabolic changes post-fasting.

Part 6: Supplementary materials of OGT experiments

Supplementary Figure 6.1. Time course from glucose CEST signals from other subjects in OGT experiments.
Supplementary Figure 6.2. All time-point images revealing well-alignment, with pancreas depicted.

Part 7: Supplementary results of liver patients

Supplementary Figure 7.1. SSE-CEST results on a patient with benign liver lesion.
Supplementary Figure 7.2. All 41 slices of APTw images using SSE-CEST 2 μT protocol (same patient in Fig. 7).
Supplementary Figure 7.3. All 41 slices of ST (3.5ppm) using SSE-CEST 0.7 μT (active tumor: hyper-intense. Necrosis: hypo-intense).
Supplementary Figure 7.4. The Z-spectral and LD-spectral comparison among active tumor, necrosis and normal-appearing control regions.
Supplementary Figure 7.5. Comparison of SSE-CEST reconstruction using different num. of adjacent slices.
Supplementary Figure 7.6. Demonstration of high-resolution SSE-CEST for depicting small lesions.

Supplementary methods

Quantification methods

1. SSE-CEST reconstruction details with calculation of the spatial-spectral resolution

Rationale for spectral reconstruction using adjacent slices: inherent tolerance for heterogeneity under partially separable theory: The idea of SSE-CEST reconstruction is similar to that used in MR spectroscopic imaging, based on a subspace model termed as Partially Separable Functions (PSF) theory, a mathematical framework for representing high-dimensional spatiotemporal data in a low-dimensional subspace. Since the time scale of single-breath-hold only allows acquiring 8-12 lines, which means each slice only sampled 8-12 frequency offsets. SSE-CEST does not assume uniform spatial contribution of CEST signals across the entire FOV or across the adjacent slices. Instead, the main purpose of putting together adjacent slices is the augmentation for sampled frequencies, ensuring that each group of voxels got 3× sampled offsets for spectral details. Briefly, we didn't directly put the value of adjacent frequencies ($\omega \pm \Delta\omega$) to a certain voxel. But for SVD feature extraction, we combined voxels within three adjacent slices, forming a matrix of $M \times 3V$, where M denoted the normalized z-ω lines and $Nvoxel = Nx \times Ny$ denotes the in-slice voxel numbers. As long as the adjacent slices containing voxels exhibiting similar spectral features (belonged to the same group), their spectral and spatial details could be reconstructed with high-fidelity. Noted that we do not perform any truncation that would discard components representing spectral or spatial details. For example, for the in vivo 2 μT protocol of human abdomen, CEST datasets from 5 reconstructed slices are put together for SVD, each with 11 distinct sampled offsets. (five sets of 11 offsets × 6400 voxels, each set with slight-shifted distinct offsets). Therefore, the resulting spectral basis functions, therefore, collectively represent the full range of spectral shapes within the grouped voxels, i.e., both normal tissue and any potential focal lesions present within the adjacent slices.

The spatial-spectral resolution: SSE-CEST does not change the acquired resolution on Z-dimension, either for the in-plane resolution. The resolution in z-ω space is intrinsically determined by the spectral-spatial encoding relationship $\Delta\omega = \gamma \cdot G_{sat} \cdot \Delta z$. Briefly, the slice thickness (Δz), namely the spatial resolution along the z-axis, is calculated as the slice-direction field of view (FOV_s) divided by the number of slices (41 used for single-breathhold liver imaging). For a fixed FOV and number of slices, the acquired spectral steps $\Delta\omega$ is directly proportional to the smallest non-zero G_{sat}. This coupling between spatial and spectral dimensions is a fundamental characteristic of the SSE-CEST encoding scheme. For instance, in the in vivo parallel-line acquisition protocol, the spatial resolution along z *(i.e.* Δz*)* is the slice thickness, given by the FOV (150 mm) divided by the number of slices (41), resulting in Δz = 3.66 mm; The spectral resolution $(\Delta\omega)$ is then determined by the smallest saturation gradient used (G_{sat} = -0.1 mT/m) and Δz. This yields a native spectral resolution of $\Delta\omega \approx 0.1$ ppm.

2. Introduction of single-pool LD quantification

Under a relatively small saturation B1 ($B_{1,sat}$ = 0.7 μT here), the contribution from direct saturation (DS) of water could be described as a Lorentzian line-shape (Zaiss et al., 2011). For a small B1,sat (e.g 0.7 μT), MTC is also small and could be either neglected (Jones, C 2012 MRM)(Jones et al., 2012) (Dula et al., 2013)or considered as a constant when Δω are close to water (e.g. -10 ppm to 10 ppm)(Deshmane et al., 2019; Desmond et al., 2014; Zaiss et al., 2011) . In the so-called LD analysis, the background Z-spectra (Z_{CEST}^{LF}) is firstly fitted as reference without CEST contribution. Then took the subtraction of Z_{CEST}^{LF} and the acquired Z-spectra (Z_{CEST}^{exp}) for CEST extraction.

$$\mathrm{LD} = \mathbf{Z_{CEST}^{LF}} - \mathbf{Z_{CEST}^{exp}} \quad \text{(Eq.1)}$$

Herein for a small B1 under 3T scanners, we fitted the Z_{CEST}^{LF} by a single-pool Lorentzian function for DS, with the contribution of MTC considered as a constant baseline:

$$Z_{CEST}^{LF} = 1 - L_{DS} - MTR_c = b - \frac{A \cdot \Gamma^2/4}{\Gamma^2/4 + \Delta\omega^2} \quad \text{(Eq.2)}$$

in which A denotes the magnitude of water line, Γ is the line-width, Δω is the frequency offset from water, and b is a baseline. Quantification based on single-pool LD methods were employed in quantifying human muscle glycogen (Bie et al., 2025), and was also preliminarily validated on human fasting experiments using a free-breathing liver imaging sequence (Xu et al., 2025), both showing good robustness. To fit in the single-breathing time scale, Our SSE-CEST separately acquired the positive part (Zpos-0.7μT) and the negative part of Z-spectra (Zneg-0.7μT), both using a saturation B1 of 0.7μT same as previous study(Xu et al., 2025). Briefly, for Zneg (0.7μT) data, we consider the Z-spectrum frequency offset ranges without CEST contributions to be [-10:-6, -0.4:0.6, 0.7:1.5] ppm; For Zpos-0.7μT data, the offset range used is [-0.6: -0.4, -0.3: 1.1, 6: 10] ppm. Fitting the Z-spectrum within these corresponding offset ranges using Eq.2 yields the Z_{CEST}^{LF}.

Noted this model neglects water line broadening and the distortions due to magnetization transfer contributions, therefore may cause inaccurate quantification for diverse tissue types and/or larger saturation B1 field. Nevertheless，owing to the highly-efficient spectral interpolation and motion-stabilizing of SSE-CEST，other refined fitting strategies could be integrated in future— including Gaussian, Voigt, or multi-component Lorentzian schemes — combined with machine-learning(Chen et al., 2023) or deep-learning (Chen et al., 2020) .

Introduction of R1pho background quantification

Alternatively, we also employed analysis based on R1ρ physical model, rather than the LD analysis base on spectral line-shape. Briefly, R1ρ describes the decay rate of longitudinal magnetization in a CEST or Spin-Lock MR experiment (Jin et al., 2012; Zaiss & Bachert, 2013). The metabolic-specific contributions, termed as exchange-dependent relaxation in the rotating frame (Rex), could be extracted by removing the R1ρ effect from background water ($R_{1\rho,w}$):

$$R_{ex} = R_{1\rho} - R_{1\rho,w} = R_{1\rho} - (R_{1w}\cos^2\theta + R_{2w}\sin^2\theta) \quad \text{(Eq.3)}$$

Where $\theta = \mathrm{atan}\left(\frac{\gamma B_1}{\Delta\omega}\right)$, for a CW-type saturation pulse employed in our SSE-CEST. The measured $R_{1\rho}$, could be calculated from the acquired Z-spectra, by

$$Z = \left(\frac{1-e^{-R_{1a}TR}}{1-e^{-R_{1a}TR}\cos(\alpha)}\cos(\alpha) - M_{SS}\right) e^{-R_{1\rho}T_S} + M_{SS} \quad \text{(Eq.4)}$$

$$\text{in which } M_{SS} = \frac{R_{1a}}{R_{1\rho}}\cos^2(\theta)$$

Noted that in Eq.4, we implemented the effect of small-flip-angle(α) gradient-echo readouts that SSE utilized. $R1\rho$ quantification was demonstrated in the fasting experiments, where the endogenous T2 relaxation could also be changed due to a reduction of glycogen ((Shizhen Chen, 2023; Yadav et al., 2014)). Since the T1 and T2 mapping sequences required by above $R1\rho$ quantification, was not available in our current protocol for patient data, we only utilized LD analysis for the 0.7 μT SSE-CEST in main text.

Supplementary tables

Supplementary Table 1.1. In vivo abdominal SSE-CEST protocols

<table>
<tr><th>Contrast maps</th><th>Acquisition protocols</th><th>Quantification Metrics</th><th>Notes</th></tr>
<tr><td>Glycogen NOE
(-1.2ppm)</td><td rowspan="2">Zneg-0.7 µT:
10 parallel z-ω lines plus S_0</td><td rowspan="3">LD analysis for fasting experiments and tumor patients;
R1ρ analysis for fasting experiments where additional T1 and T2 maps were acquired.</td><td rowspan="3">Single-pool LD analysis were utilized for all 0.7 µT data from SSE-CEST and from conventional CEST, with both good image quantity and spectral profile.</td></tr>
<tr><td>Aliphatic NOE
(-3.5ppm)</td></tr>
<tr><td>Amide CEST
(3.5ppm)</td><td>Zpos-0.7 µT:
10 parallel z-ω lines plus S_0</td></tr>
<tr><td>APTw</td><td rowspan="2">Z-2 µT:
11 parallel z-ω lines plus S_0</td><td>MTRasym (3.5ppm)</td><td>Recommended by 3T clinical consensus for glioma.</td></tr>
<tr><td>GlyganCEST (GlucoCEST)</td><td>MTRasym (1.2ppm)</td><td>Often used for faster hydroxyl exchange.</td></tr>
</table>

*OGTT and the comparison with PET used 2.1 ppm for 2 µT protocol as suggested by the spectra.

Supplementary Table 1.2. Quantitative Bland-Altman results in ex vivo porcine liver in Fig. 2.

mean difference (%)			
	**Amide CEST**	**Glycogen NOE**	**Aliphatic NOE**
**SSE-parallel vs. conventional**	-0.22	-0.60	-0.33
**SSE-diamond vs. conventional**	-0.17	-0.61	-0.32
**SSE-radial vs. conventional**	-0.29	-0.03	-1.06
**95% Limits of Agreement (%)**			
	**Amide CEST**	**Glycogen NOE**	**Aliphatic NOE**
**SSE-parallel vs. conventional**	[-0.71, 0.28]	[-2.58, 1.37]	[-1.10, 0.44]
**SSE-diamond vs. conventional**	[-0.74, 0.39]	[-2.36, 1.15]	[-1.08, 0.44]
**SSE-radial vs. conventional**	[-1.32, 0.74]	[-2.21, 2.14]	[-2.63, 0.51]

*B1 = 0.7 µT, (n = 69, the number of liver ROIs (8×8 square) within the displayed slice).

Supplementary Table 1.3. Quantitative results of correlation coefficients in healthy volunteers in Fig. 4b.

	sub. 1	sub. 2	sub. 3	sub. 4	sub. 5	sub. 6	sub. 7	sub. 8	sub. 9	sub. 10
correlation coefficients										
ST (1.2 ppm)	0.9480	1.0000	0.9636	0.9985	0.9996	0.8986	0.7121	0.7729	0.9908	0.8545
ST (3.5 ppm)	0.8347	0.9998	0.9980	0.9973	0.9638	0.9133	0.9030	0.7975	0.9939	0.9671
ST (-3.5 ppm)	0.8399	0.9996	0.9758	0.9951	0.9946	0.8148	0.6000	0.1839	0.9945	0.9394
APTw	0.7810	0.9999	0.9997	0.9977	1.0000	0.8684	0.6708	0.8103	0.9945	0.9552
p values										
ST (1.2 ppm)	<0.0001	<0.0001	<0.0001	<0.0001	<0.0001	0.0004	0.0209	0.0088	<0.0001	0.0035
ST (3.5 ppm)	0.0027	<0.0001	<0.0001	<0.0001	<0.0001	0.0002	0.0009	0.0057	<0.0001	<0.0001
ST (-3.5 ppm)	0.0024	<0.0001	<0.0001	<0.0001	<0.0001	0.0041	0.0731	0.6110	<0.0001	<0.0001
APTw	0.0077	<0.0001	<0.0001	<0.0001	<0.0001	0.0011	0.0337	0.0045	<0.0001	<0.0001

Supplementary Table 1.4. Quantitative results of normalized mean squared error in healthy volunteers in Fig. 4c.

	sub. 1	sub. 2	sub. 3	sub. 4	sub. 5	sub. 6	sub. 7	sub. 8	sub. 9	sub. 10
normalized mean squared error (%)										
ST (1.2 ppm)	0.1792	0.0044	0.0497	0.0233	0.0014	0.2359	0.1400	0.1685	0.0134	0.0202
ST (3.5 ppm)	0.0460	0.0009	0.0129	0.0838	0.0486	0.2071	0.1046	0.3198	0.0273	0.0841
ST (-3.5 ppm)	0.0279	0.0009	0.0581	0.0529	0.0330	0.3739	0.1290	0.2415	0.0240	0.1025
APTw	0.0180	<0.0001	0.0452	0.0309	0.0156	0.1667	0.0244	0.0783	0.0034	0.0184

Supplementary figures

Part 1: Reconstruction basis of SSE-CEST framework and its compatibility

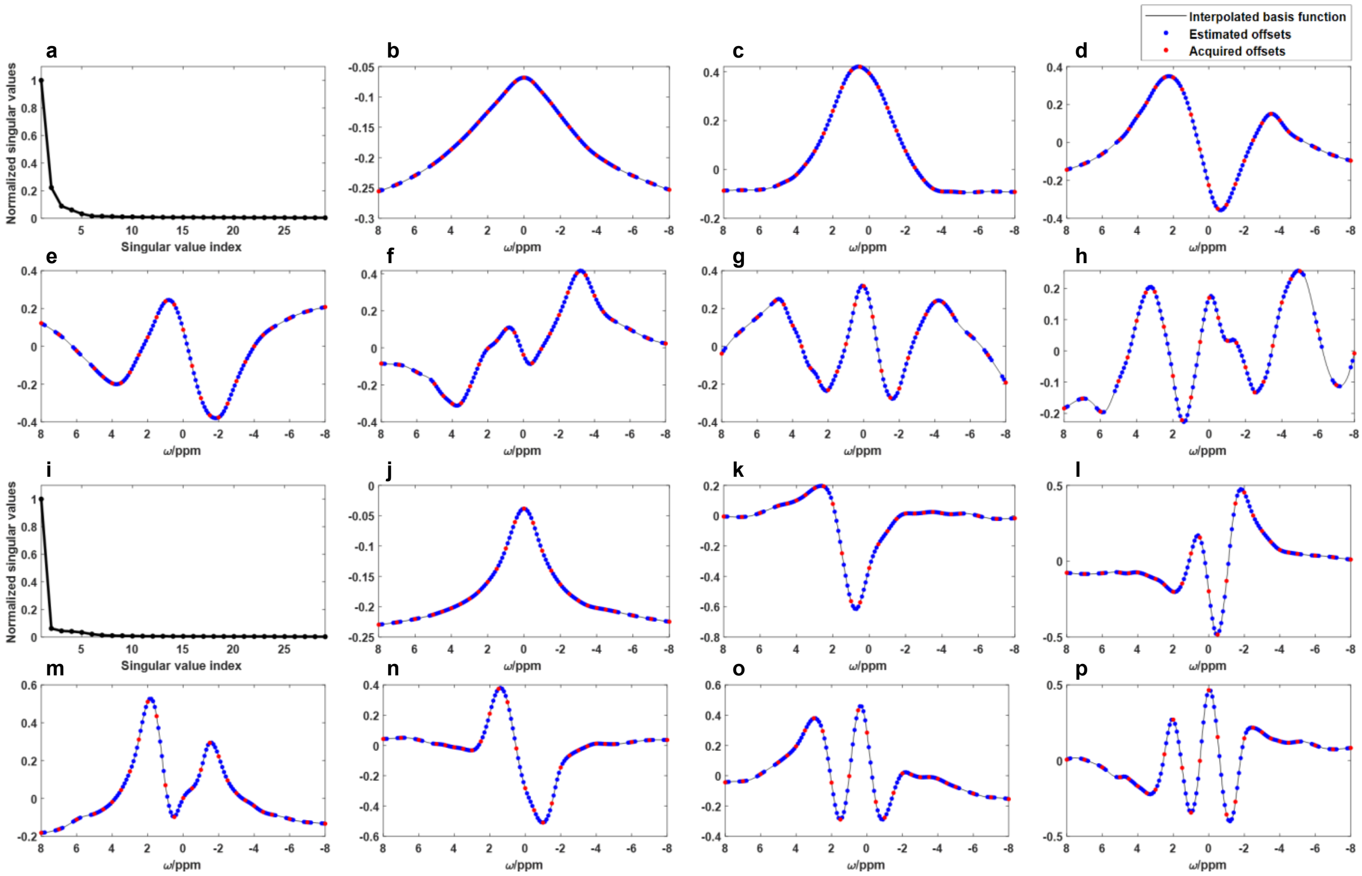

Supplementary Figure 1.1. Spectral bases of adjacent slices from ex vivo porcine liver. a, Normalized singular value (2 μT) indicates the low-rankness of CEST spectra, with the normalized values decaying exponentially and reaching a weight closing to zero after 7th (<0.02). **b**,**c**,**d**,**e**,**f**,**g**,**h**, The first to seventh spectra basis sorted by the singular value (2 μT), with dots indicating the sampling ω of SSE-CEST and the solid line is the polynomial interpolation. **The red dots: the reconstructed slice; The blue dots: 4 adjacent slices (2 acquired slices with 2X reconstruction). i**, Normalized singular value (0.7 μT). **j**,**k**,**l**,**m**,**n**,**o**,**p**, The first to seventh spectra basis sorted by the singular value (0.7 μT).

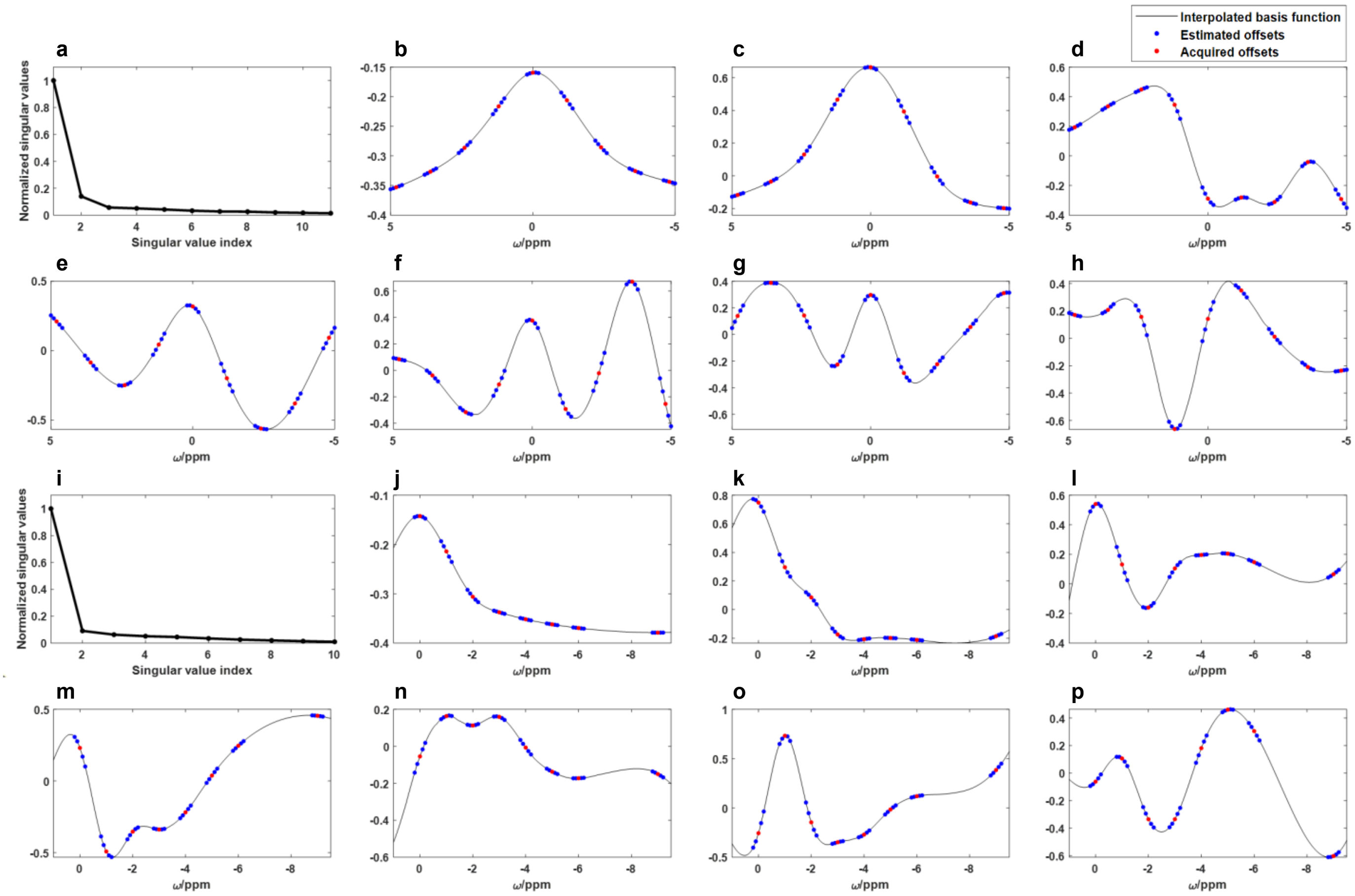

Supplementary Figure 1.2. Spectral bases extracted from SSE-CEST on a human subject liver. a, Normalized singular value (2 μT) indicates the low-rankness of CEST spectra, with the normalized values decaying exponentially and reaching a weight closing to zero after 7th (<0.03). **b**,**c**,**d**,**e**,**f**,**g**,**h**, The first to seventh spectra basis sorted by the singular value (2 μT), with dots indicating the sampling ω of SSE-CEST and the solid line is the polynomial interpolation. **The red dots: the reconstructed slice; The blue dots: four adjacent slices (2 acquired slices with 2X reconstruction) i**, Normalized singular value (0.7 μT). **j**,**k**,**l**,**m**,**n**,**o**,**p**, The first to seventh spectra basis sorted by the singular value (0.7 μT).

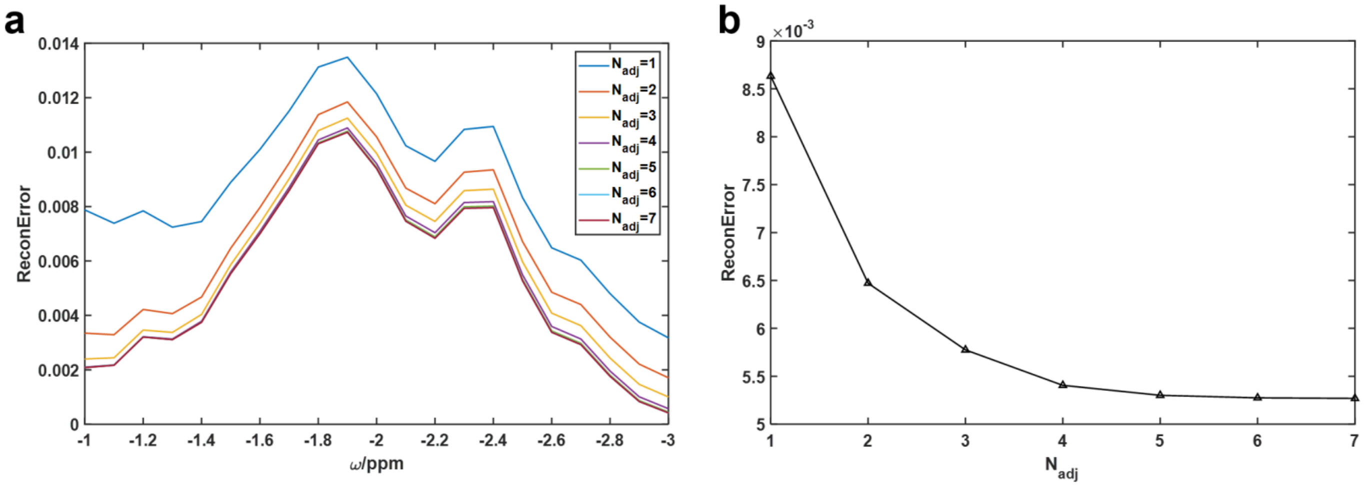

Supplementary Figure 1.3. Influence of the number of adjacent slices on the reconstruction accuracy in ex vivo porcine liver. Reconstruction accuracy was quantified as the voxel-wise absolute difference between the reconstructed data and the reference (gold-standard) CEST acquisition. **a**, Reconstruction error plotted across frequency offsets (ω) when different numbers of adjacent slices (Nadj) are included in the spectral-basis interpolation. **b**, Mean reconstruction error as a function of the total number of adjacent slices used in the interpolation. The error decreases as more adjacent slices are incorporated and plateaus when the adjacent slice number reaches five—the configuration adopted for all reconstructions in this study—

indicating that the interpolation is robust and that additional slices provide diminishing improvements. Note that the number of adjacent slices includes the target slice itself.

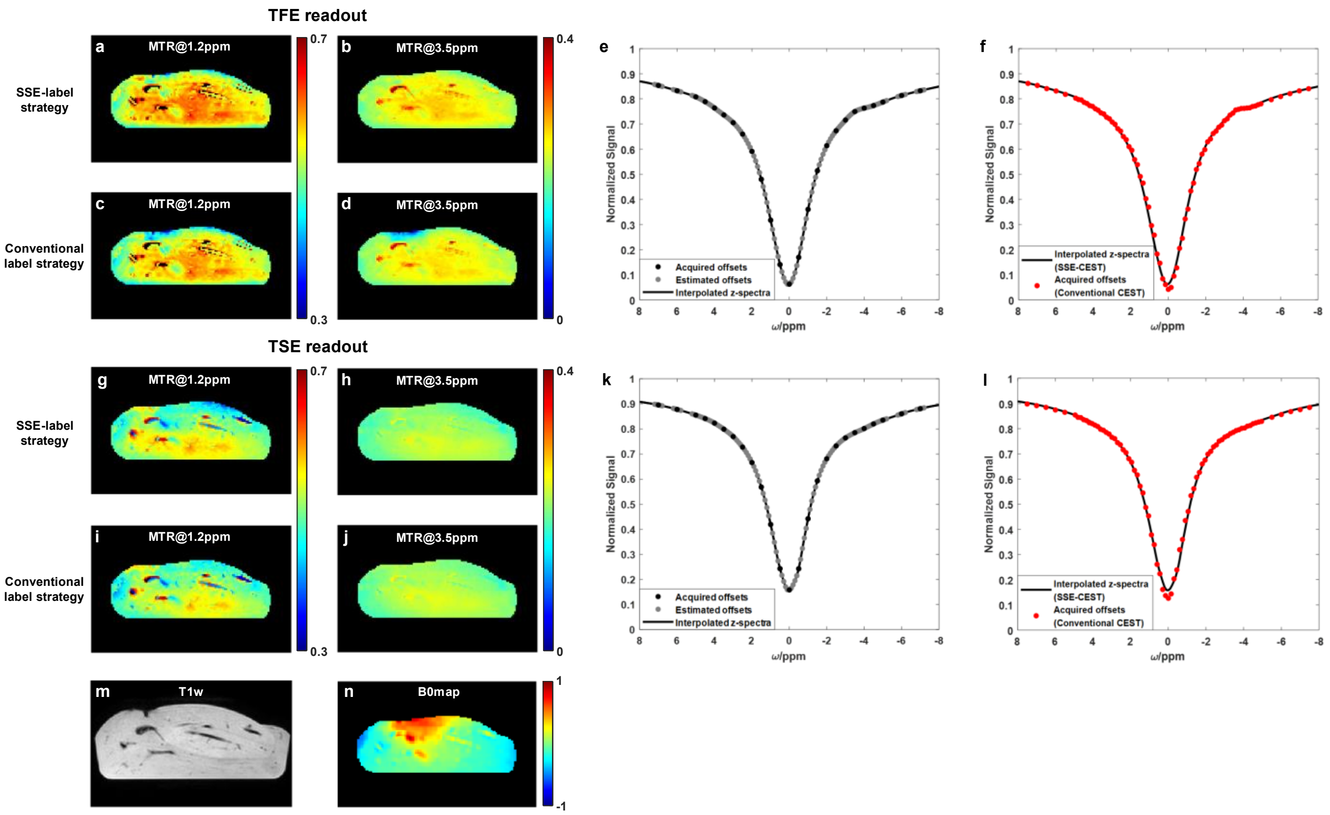

Supplementary Figure 1.4. Flexibility of SSE-CEST with respect to readout types validated on ex-vivo porcine liver. For TFE-readout. **a**,**b**,**c**,**d**, MTR maps (or so-called ST maps) from SSE-CEST and conventional CEST using **e**,**f**, comparison of z-spectra from SSE-CEST and from conventional CEST using TFE-readout. **g**,**h**,**i**,**j**, MTR maps from SSE-CEST and conventional CEST using TSE-readout. **k**,**l**, comparison of z-spectra from SSE-CEST and conventional CEST using TSE-readout. **m**, the T1 weighted image of the porcine liver anatomical structure. **n**, B0 shift distribution.

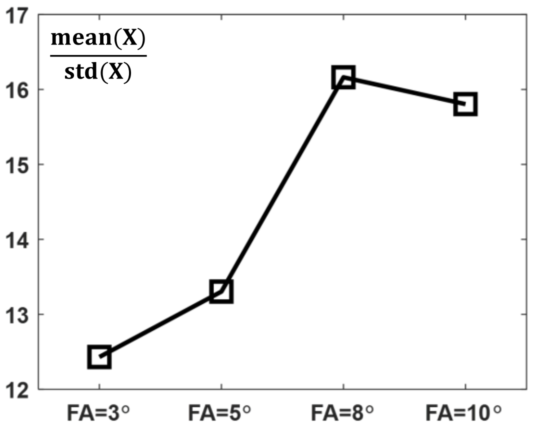

Supplementary Figure 1.5. Optimization of the readout flip-angles for human subjects. According to the value of liver parenchyma that calculated by mean intensity divided by the standard deviation, the flip angle was fixed to 8°, to improve the image contrast.

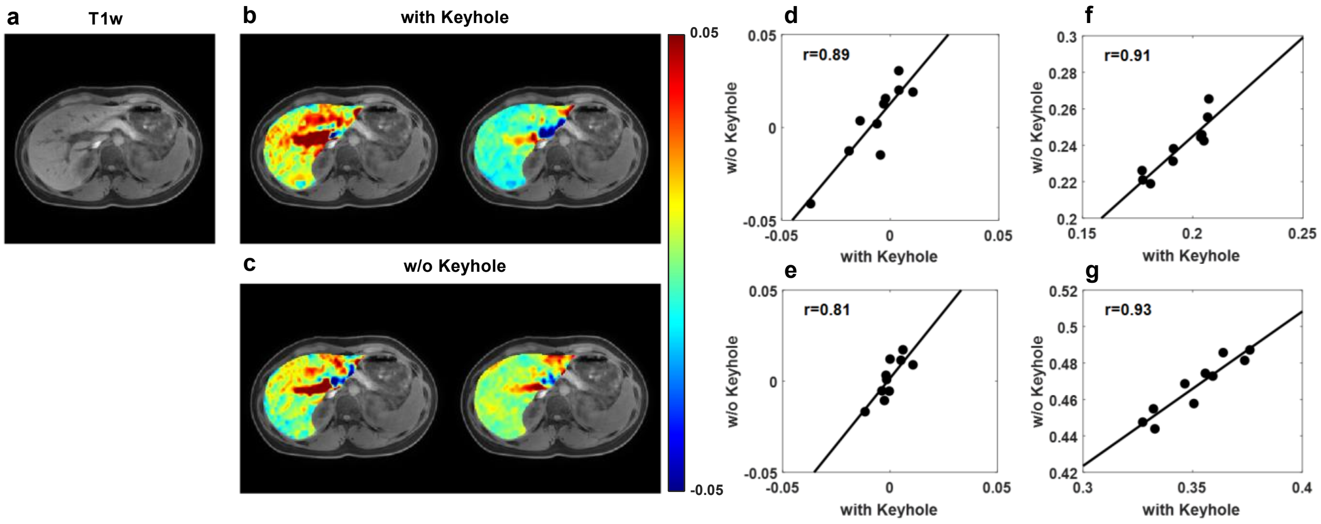

Supplementary Figure 1.6. Data from a healthy volunteer that demonstrates the combination of SSE-CEST and 'Keyhole' technique. a, The T1 weighted image. **b**, MTR asymmetry maps at 3.5 ppm and 1.2 ppm, with 50% keyhole under-sampling. **c**, MTR asymmetry maps at 3.5 ppm and 1.2 ppm without keyhole under-sampling. **d**,**e**, Pearson correlation analysis on normalized saturation images. **f**,**g**, Pearson correlation analysis on MTR asymmetry maps.

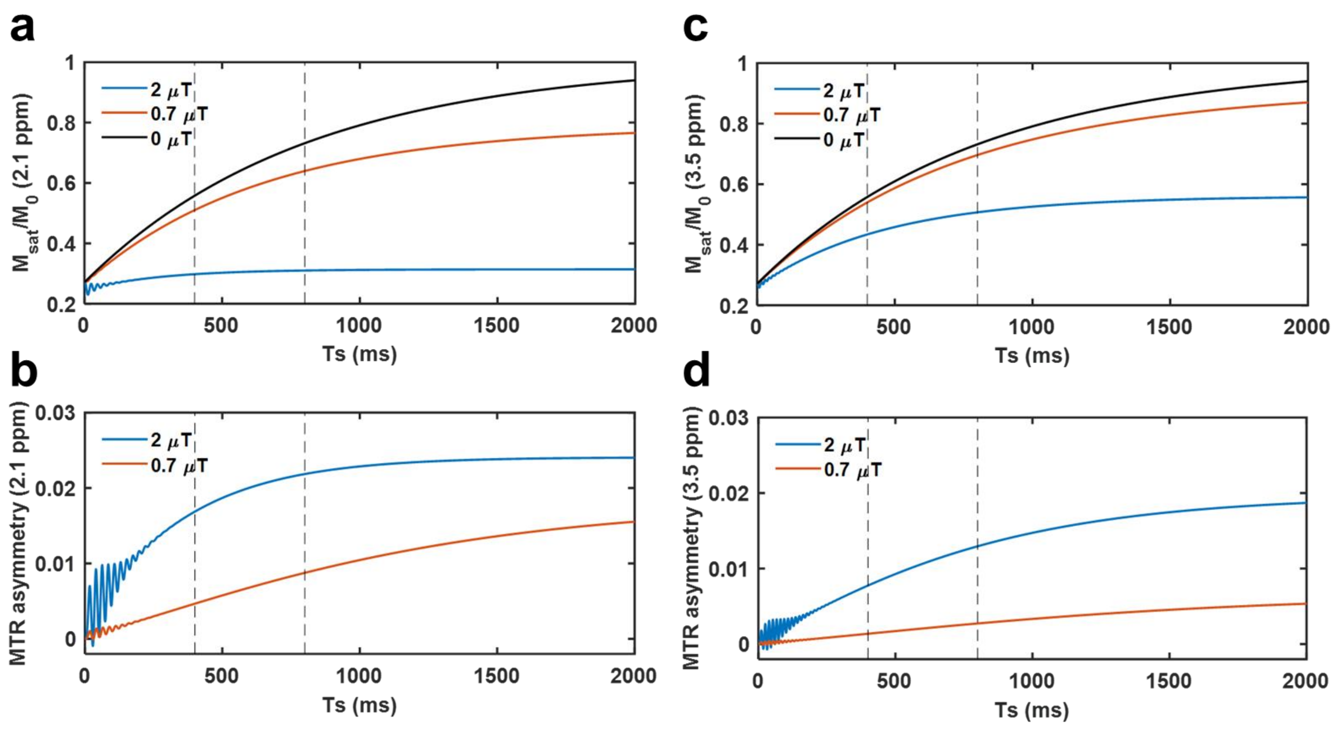

Supplementary Figure 1.7. Numerical simulation of CEST signal evolution for fast- and slow-exchange metabolites under SSE-CEST conditions. The Bloch-McConnell simulation includes five pools for water, amide, and three hydroxyl groups. (**a**) Msat/M0 signal evolution at 2.1 ppm (representing fast-exchange pools such as hydroxyls) and (**c**) at 3.5 ppm (representing the slower-exchange amide pool) as a function of saturation time (Tsat). The initial magnetization started from the readout steady-state (Mi =0.272), reflecting the zero-recovery-time design of the SSE-CEST sequence, rather than from thermal equilibrium (M0=1). (b, d) The corresponding MTRasym dynamics at 2.0 ppm and 3.5 ppm, respectively. At the chosen in vivo Tsat, the transient R2ρ oscillations are fully dampened, and the achieved MTRasym represents a substantial fraction of the 2s-reference signal: 73.9% (2 μT) and 57.2% (0.7 μT) at 2.0 ppm, and 41.6% (2 μT) and 51.2% (0.7 μT) at 3.5 ppm.

Part 2: Ex-vivo porcine liver experiments

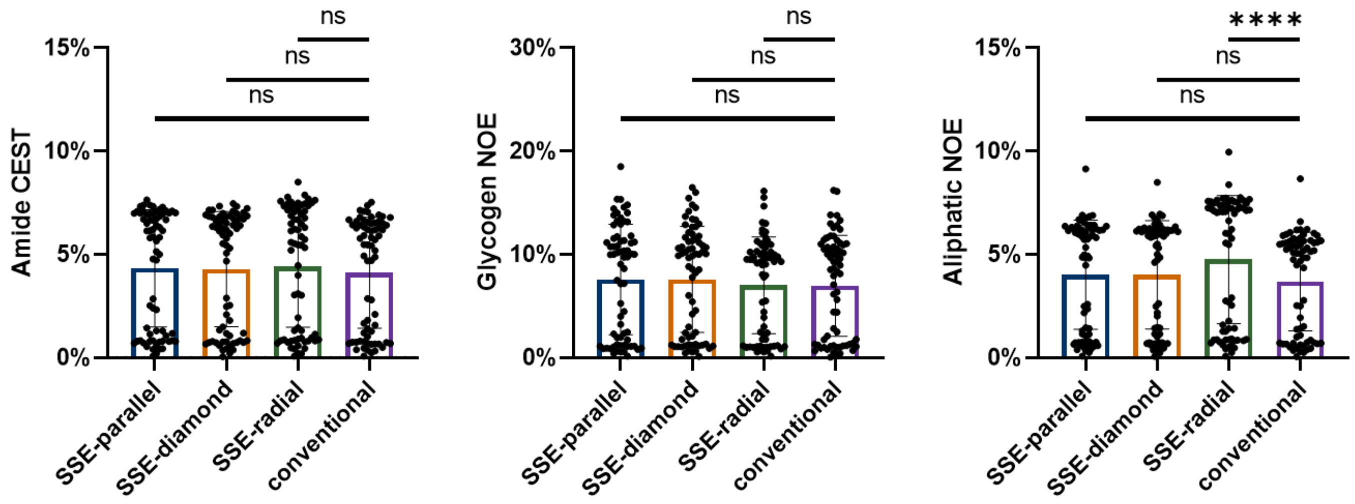

Supplementary Figure 2.1. Quantitative comparison of CEST contrast maps between SSE-CEST and conventional CEST in ex vivo porcine liver. (a) Amide CEST, **(b)** Glycogen NOE, and **(c)** Aliphatic NOE. Results from SSE-CEST with parallel, diamond, and radial z-ω acquisition patterns are displayed. All data were acquired with B1 = 0.7 μT and quantified using LD. Data are presented as mean ± SD with individual values (n = 69, the number of liver ROIs (8×8 square) within the displayed slice). One-way ANOVA was used for statistics and multiple comparison correction was done. **ns** represents p > 0.05 and **** represents p < 0.0001.

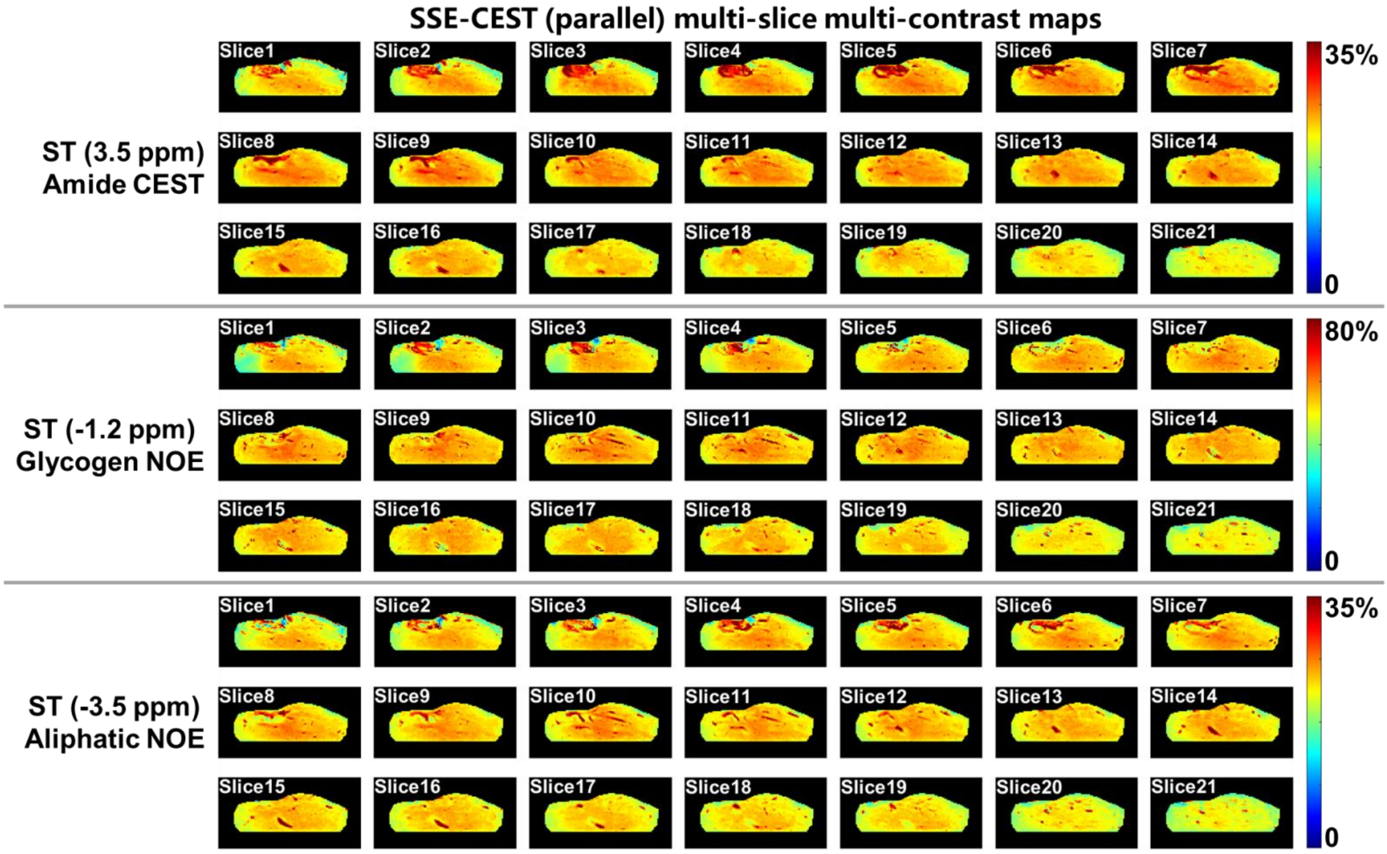

Supplementary Figure 2.2. Overview of slice variation for SSE-CEST with parallel z-ω acquisition pattern in ex vivo porcine liver. (a) Amide CEST, **(b)** Glycogen NOE, and **(c)** Aliphatic NOE. A total of 21 slices are shown. All data were acquired with B1 = 0.7 μT and quantified using ST.

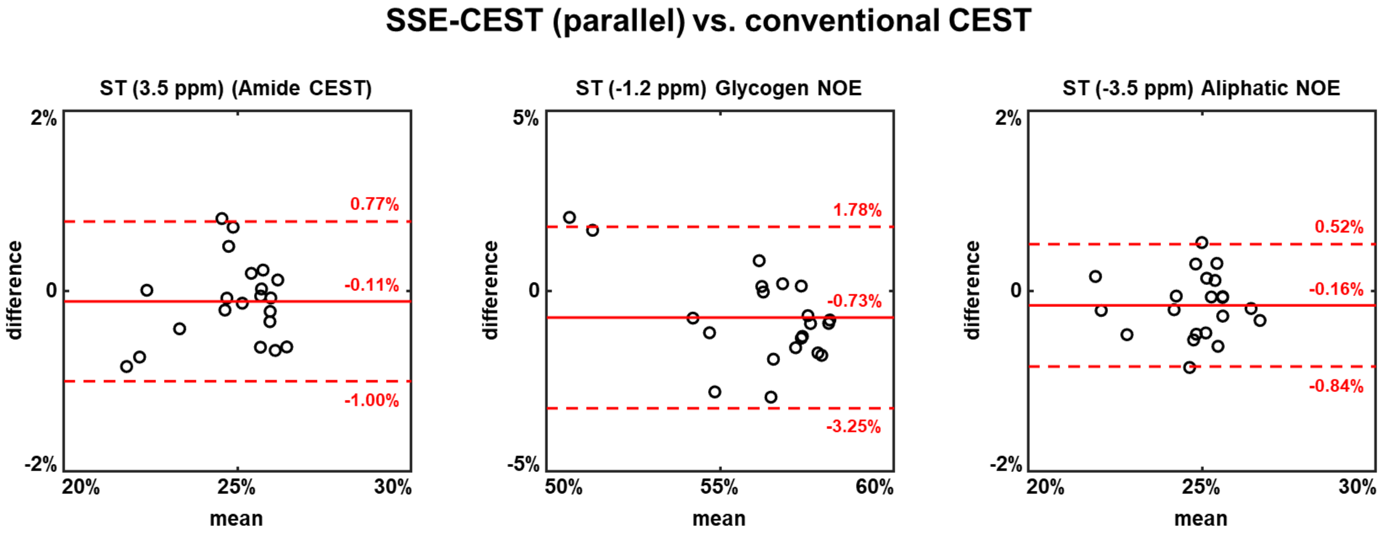

Supplementary Figure 2.3. Bland-Altman analysis between the mean signal on all 21 slices acquired by SSE-CEST and by conventional CEST (parallel z-ω acquisition pattern 0.7 μT, ex vivo porcine liver). (a) Amide CEST, **(b)** Glycogen NOE, and **(c)** Aliphatic NOE. Each circle denotes the average value of a ROI as denote in Fig. 2d (n = 21), one ROI per slice across 21 slices. Mean difference in solid lines, with the 95% Limits of Agreement in dashed lines indicating the small variance among slices.

Part 3: Glycogen phantom experiments

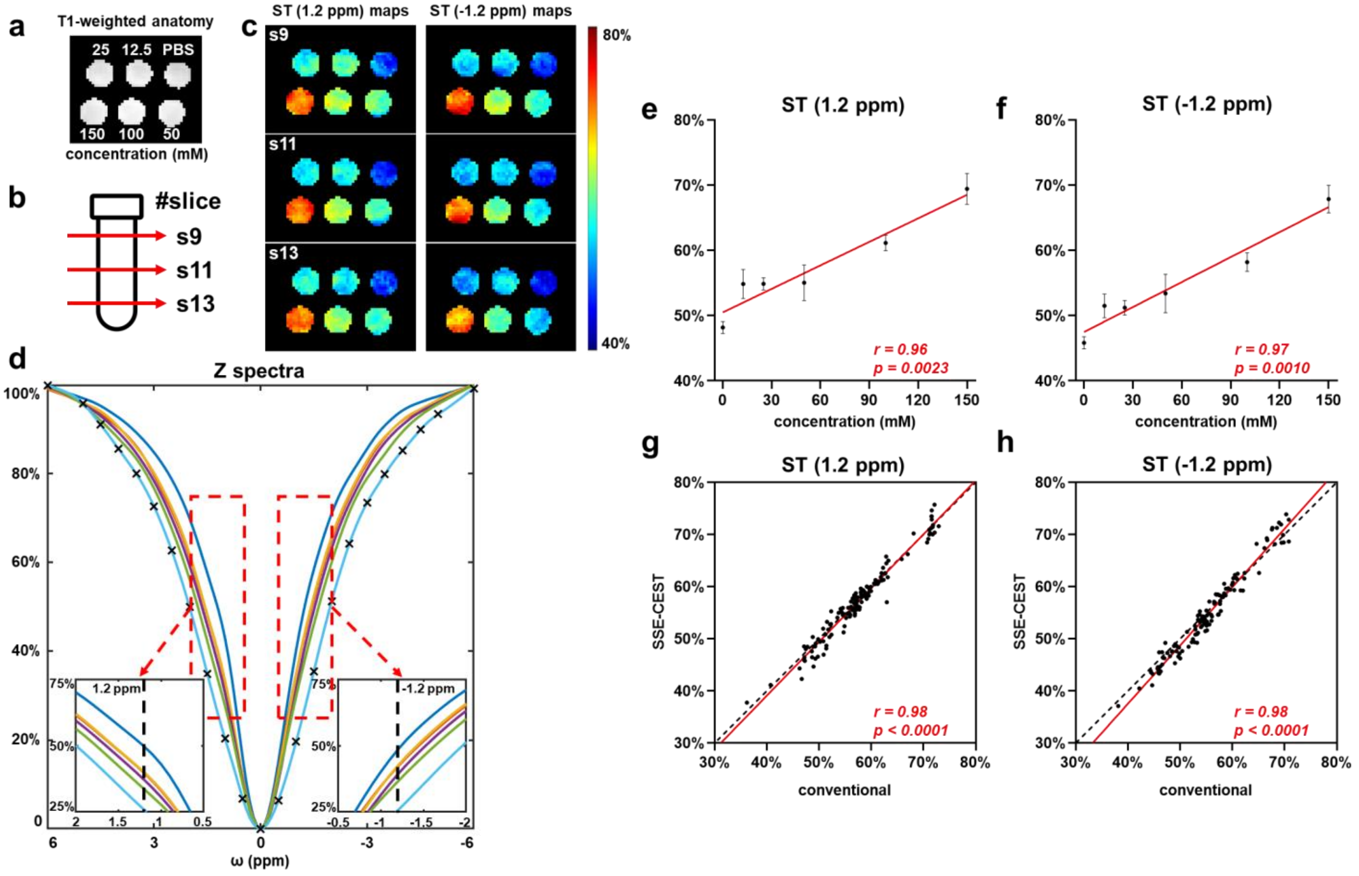

Supplementary Figure 3.1. Glycogen phantom experiments: same layout as Fig. 3 but with saturation B1 of 2 μT. a, phantom layout in x-y slices**. b,** illustration of different slices along z direction. **c,** ST (1.2 ppm) images for 'labeling' hydroxyl CEST on glycans and ST (-1.2 ppm) images for 'labeling' the replayed NOE from macromolecular glycogen respectively; with slices# 3, 6, 9, displayed. **d.** the Z-spectra of each tube on slice 11, illustrating the featured frequency range for glycan-CEST and glycogen-rNOE; **e,f,** concentration dependence of ST (1.2ppm) and ST (-1.2ppm) from SSE-CEST (n = 21 slices per tube, mean ± std, two-sided Pearson correlation) **g,h**, the scattering plot of glycan-CEST (**h**) and glycogen-rNOE (**g**) values, comparison between conventional methods and the parallel-encoded SSE methods. Each point indicates a tube ROI (6 ROIs × 21 slices, n=126, two-sided Pearson correlation).

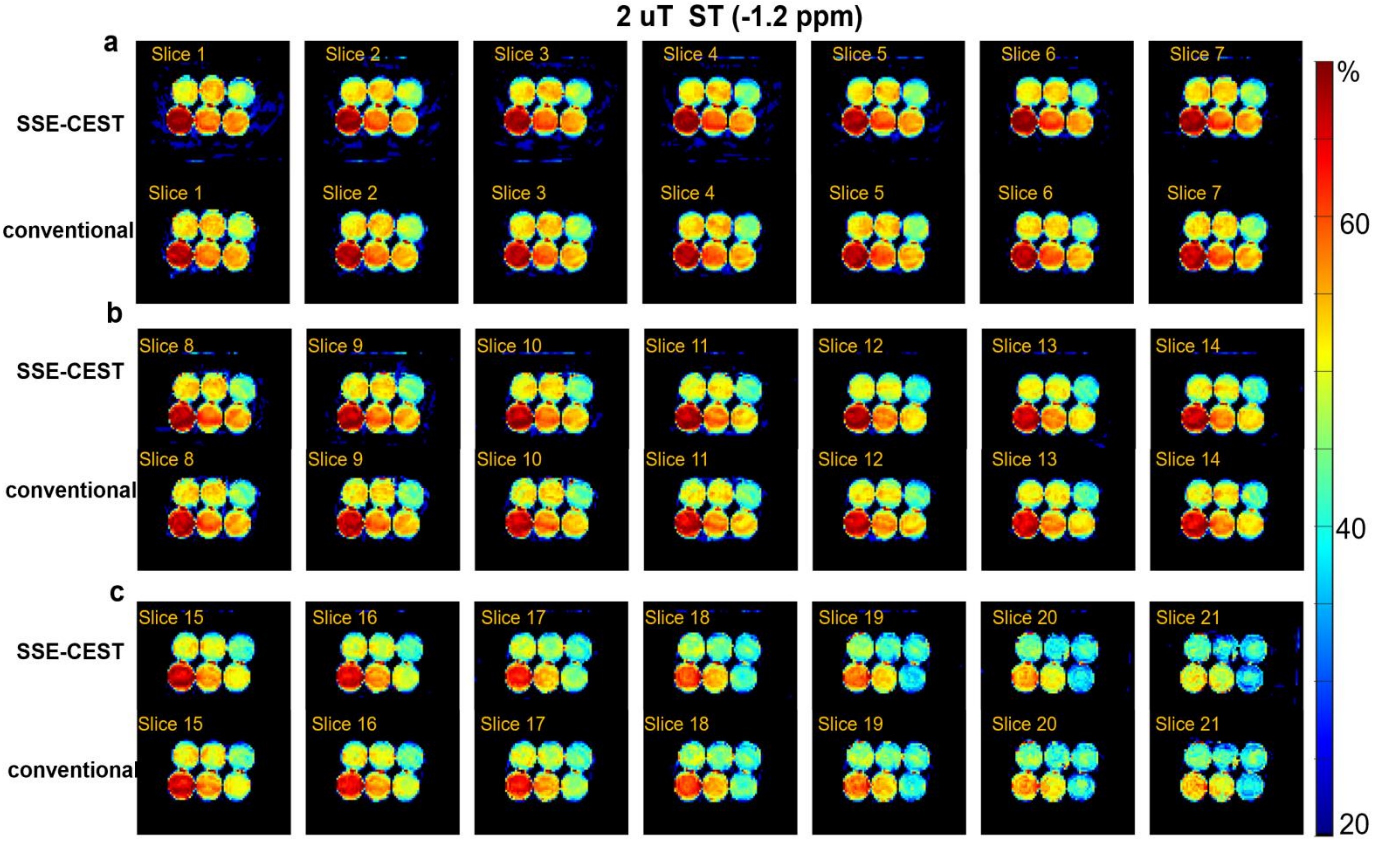

Supplementary Figure 3.2. Slice-by-slice comparison ST (-1.2 ppm) images in glycogen phantoms in 1% agar, acquired using SSE-CEST and using conventional CEST, both with a B1 of 2 μT.

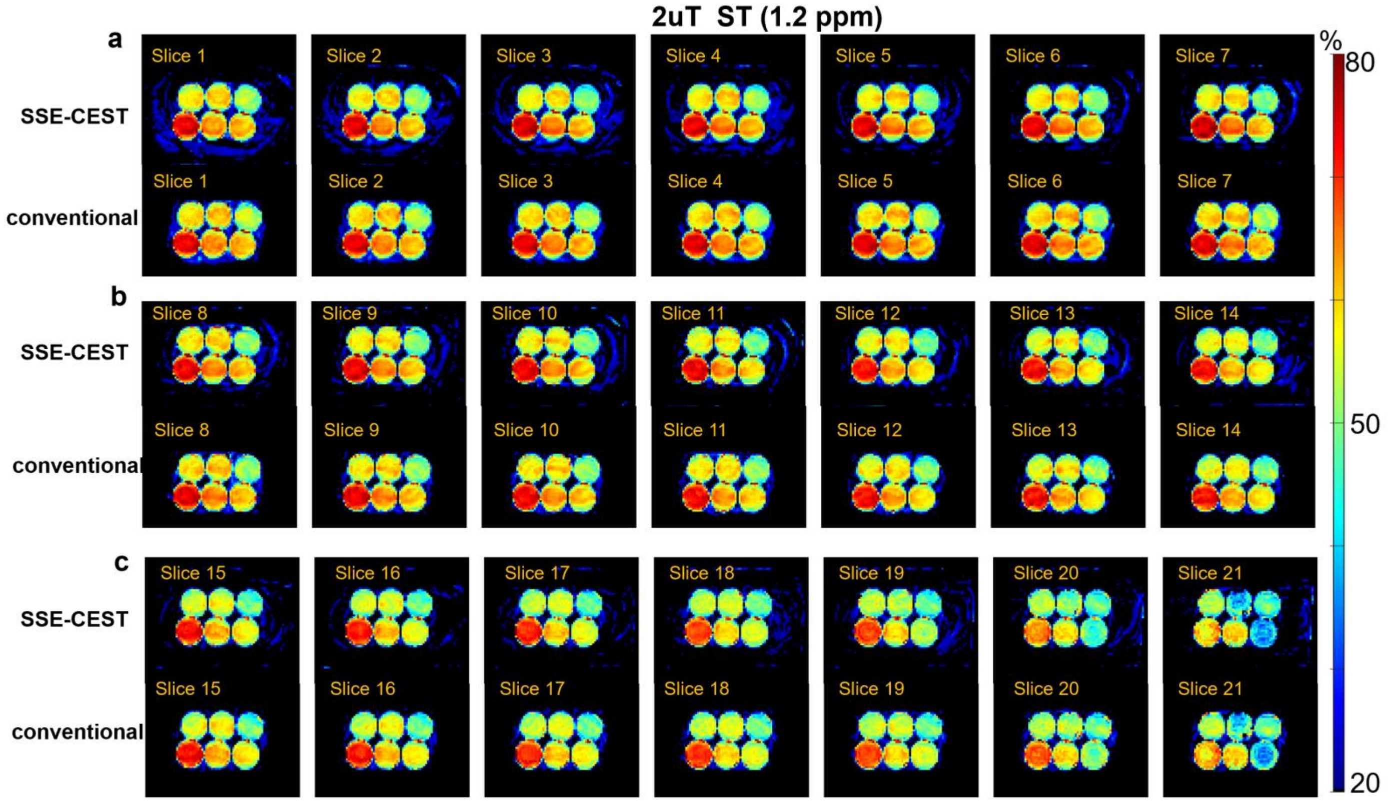

Supplementary Figure 3.3. Slice-by-slice comparison of ST (1.2ppm) images in glycogen phantoms in 1% agar, acquired using SSE-CEST and using conventional CEST, both with a B1 of 2 μT.

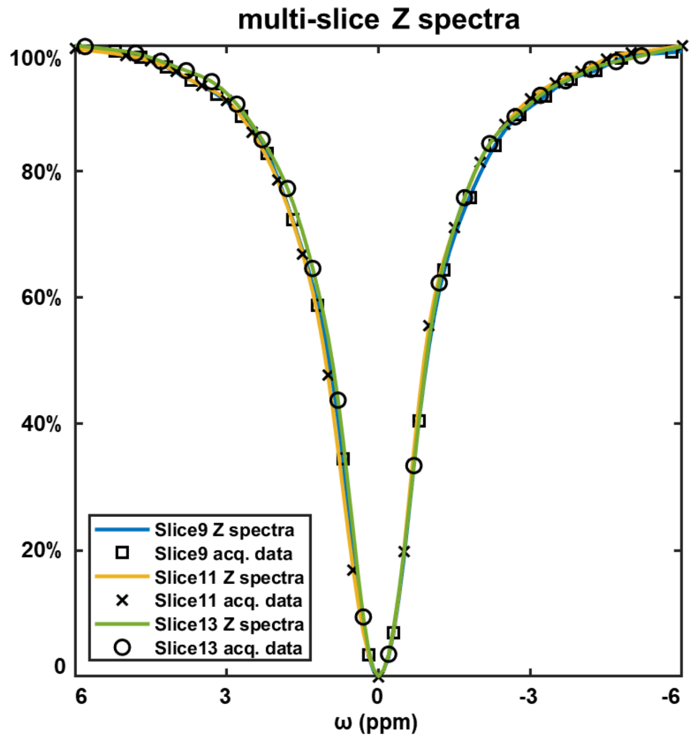

Supplementary Figure 3.4. The Z-spectra of three slices for the 150 mM glycogen tubes in main Fig. 3, with three types of markers denoting their distinct sampled ω. As seen, the reconstructed spectra are almost identical, with the slight difference due to the inhomogeneity when crosslinked with 1% agar. Also see the slice-by-slice comparison with conventional CEST in Supplementary Figs. 3.2-3.3.

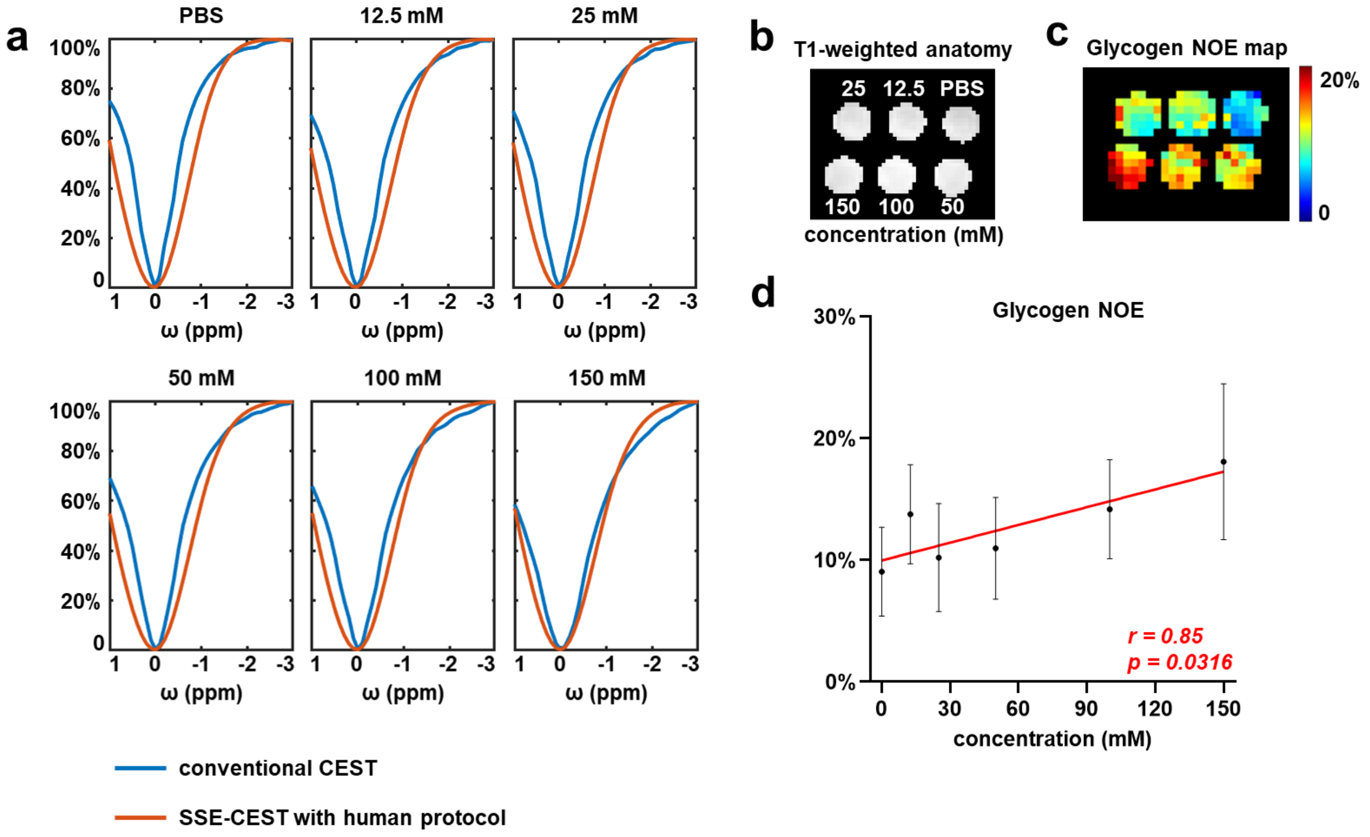

Supplementary Figure 3.5. Results of the glycogen phantom using SSE-CEST with the human acquisition protocol. a, comparison of Z spectra from conventional CEST and SSE-CEST with the human acquisition protocol. **b,** phantom layout in

x-y slices. **c,** representative glycogen NOE map of an axial slice from SSE-CEST. **d,** concentration dependence of glycogen NOE from SSE-CEST (n = 21 slices per tube, mean ± std, two-sided Pearson correlation). All data were acquired with B1 = 0.7 μT and quantified using LD. Representative parameters: conventional CEST: Tsat/TR = 3/10 s; SSE-CEST: Tsat/TR = 0.8/1.87 s.

Part 4: Healthy volunteers

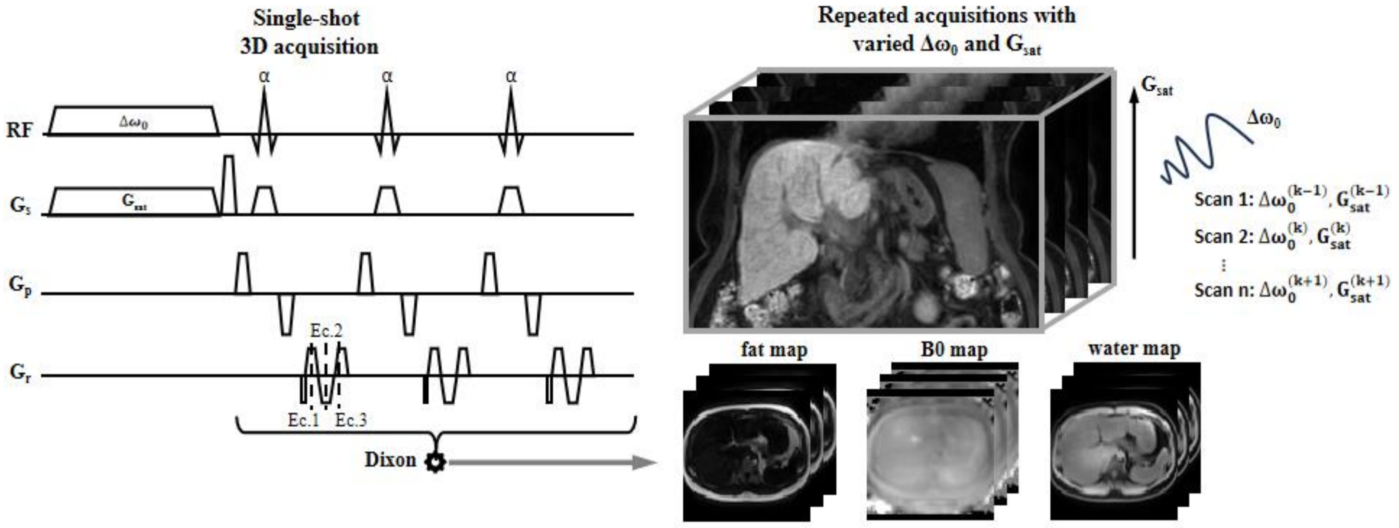

Supplementary Figure 4.1. Dixon-type 3D gradient-echo acquisition sequence for human abdominal SSE-CEST

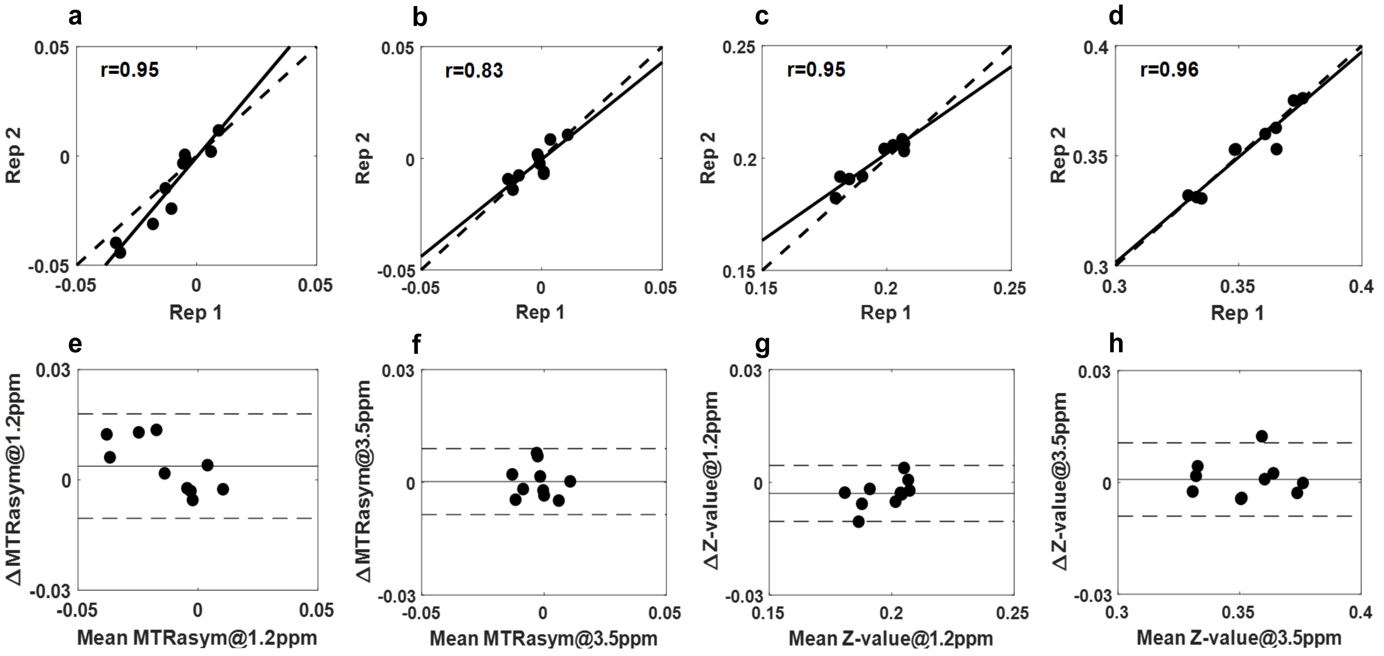

Supplementary Figure 4.2. Repeatability analysis of SSE-CEST. a,**b**,**c**,**d**, Pearson correlation of the two repeated scans. **e**,**f**,**g**,**h**, Corresponding Bland-Altman plot.

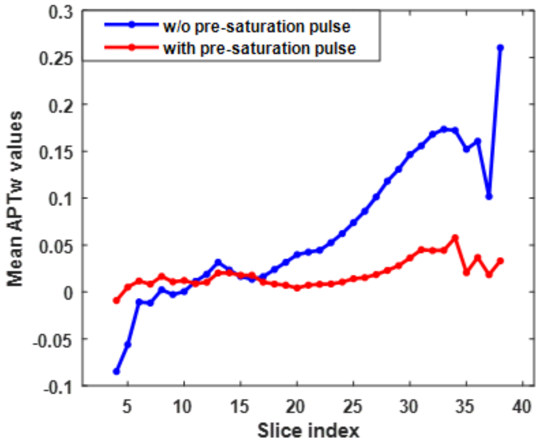

Supplementary Figure 4.3. Mean APTw values of liver parenchyma from all imaging slices. The blue line: mean APTw values of all slices without pre-saturation pulse. The red line: mean APTw values of all slices with pre-saturation pulse applied before each frequency offset.

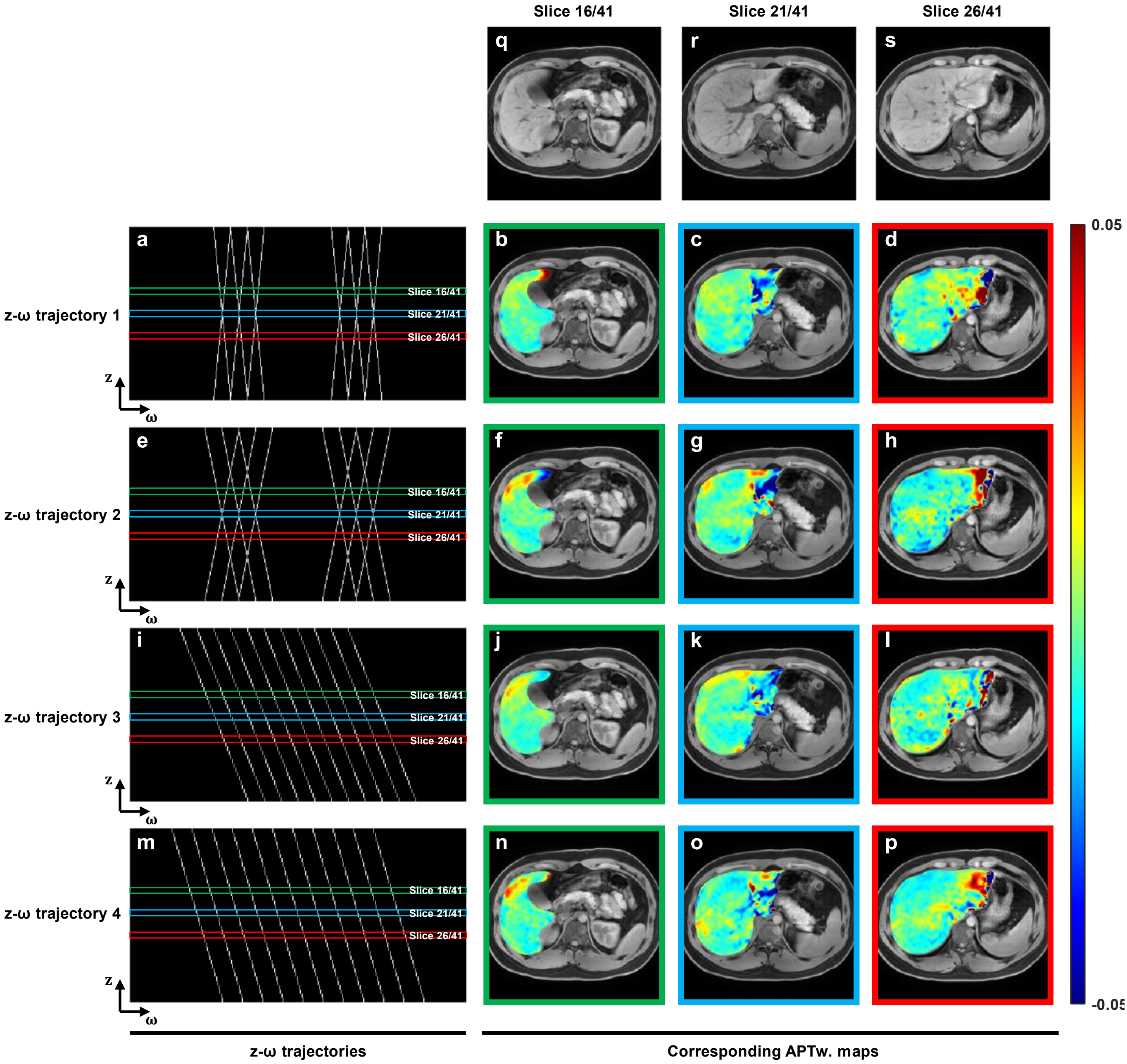

Supplementary Figure 4.4. APTw maps of multiple slices (slice 16, 21, and 26 of 41) from four designated z-ω trajectories. **a**, z-ω trajectory 1 that consists of 13 encodings. **e**, z-ω trajectory 2 that consists of 13 encodings. **i**, z-ω trajectory 3 that consists of 12 encodings. **m**, z-ω trajectory 4 that consists of 12 encodings. **b**,**c**,**d**,**f**,**g**,**h**,**j**,**k**,**l**,**n**,**o**,**p**, corresponding APTw maps. **q**,**r**,**s**, T1 weighted images of the three slices.

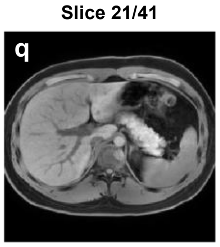

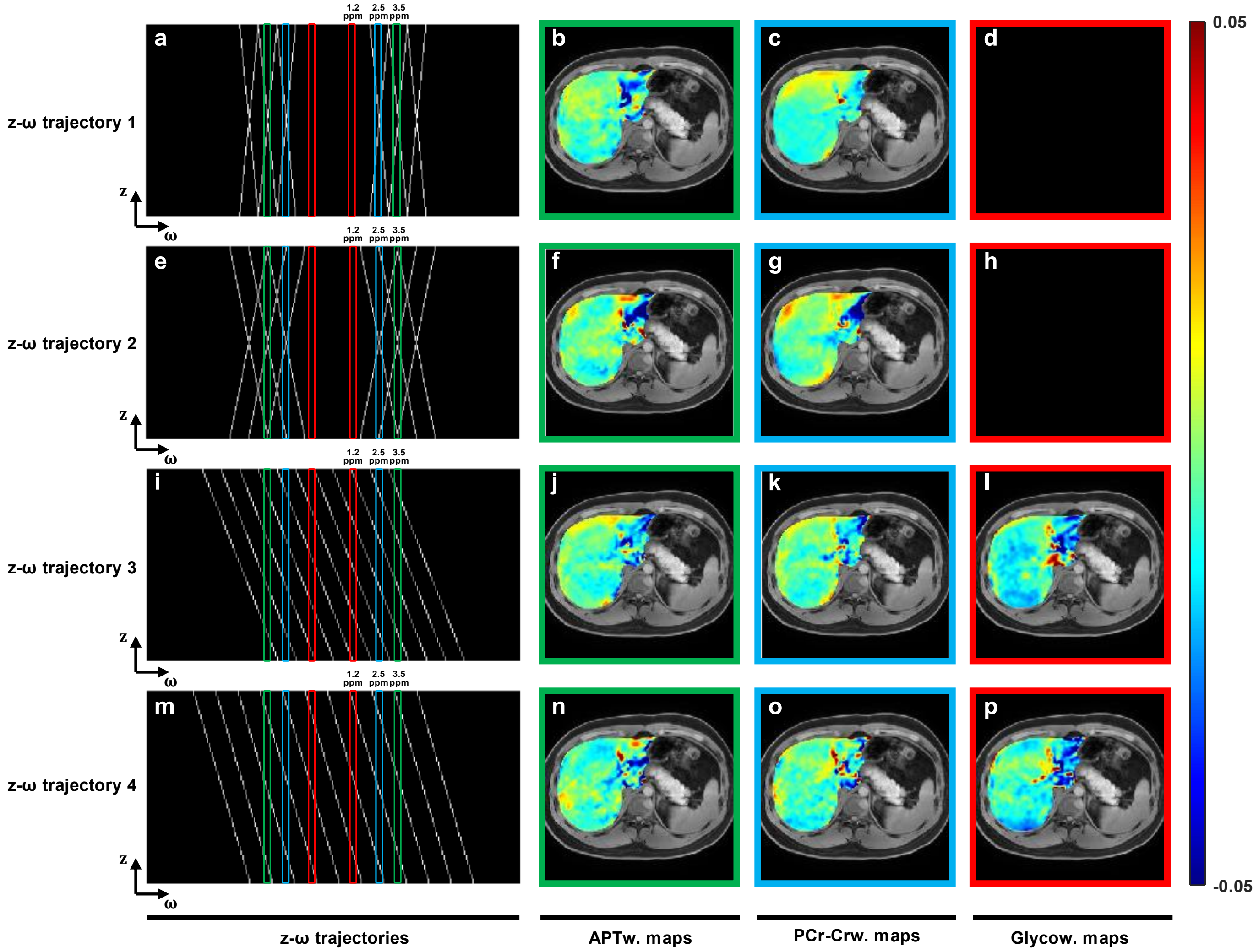

Supplementary Figure 4.5. Contrast maps of multiple metabolites from four designated z-ω trajectories. **a**,**e**,**i**,**m**, z-ω trajectory 1-4. **b**,**c**,**d**,**f**,**g**,**h**,**j**,**k**,**l**,**n**,**o**,**p**, Corresponding contrast maps. Green box: APTw maps; Blue box: Cr maps; Red box: glycogen weighted maps.

Part 5: Fasting experiments

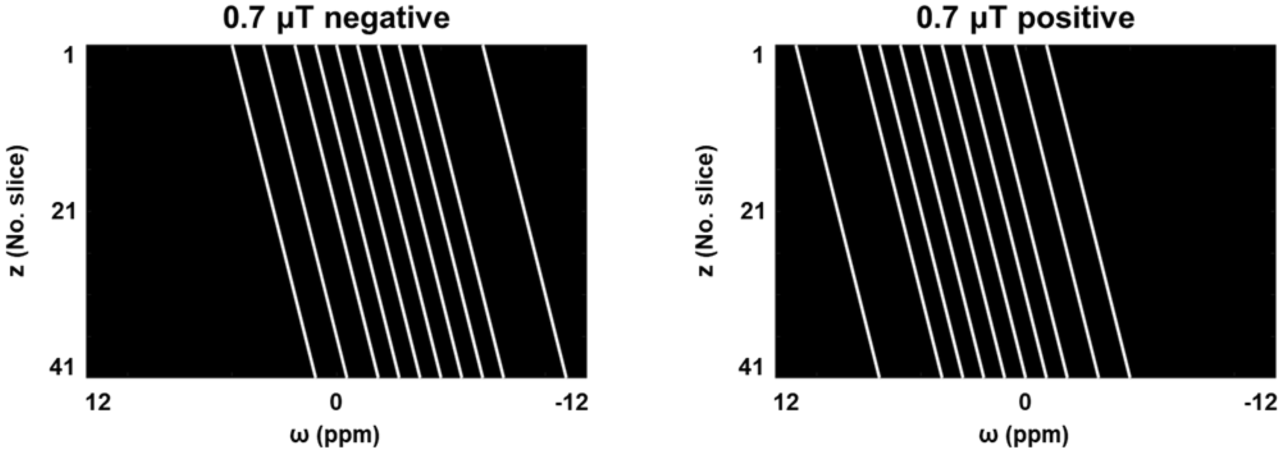

Supplementary Figure 5.1. The trajectories of Zneg (0.7 μT) and Zpos (0.7 μT) for fasting experiments and tumor patients. a, the trajectories of Zneg (0.7 μT). **b**, the trajectories of Zpos (0.7 μT).

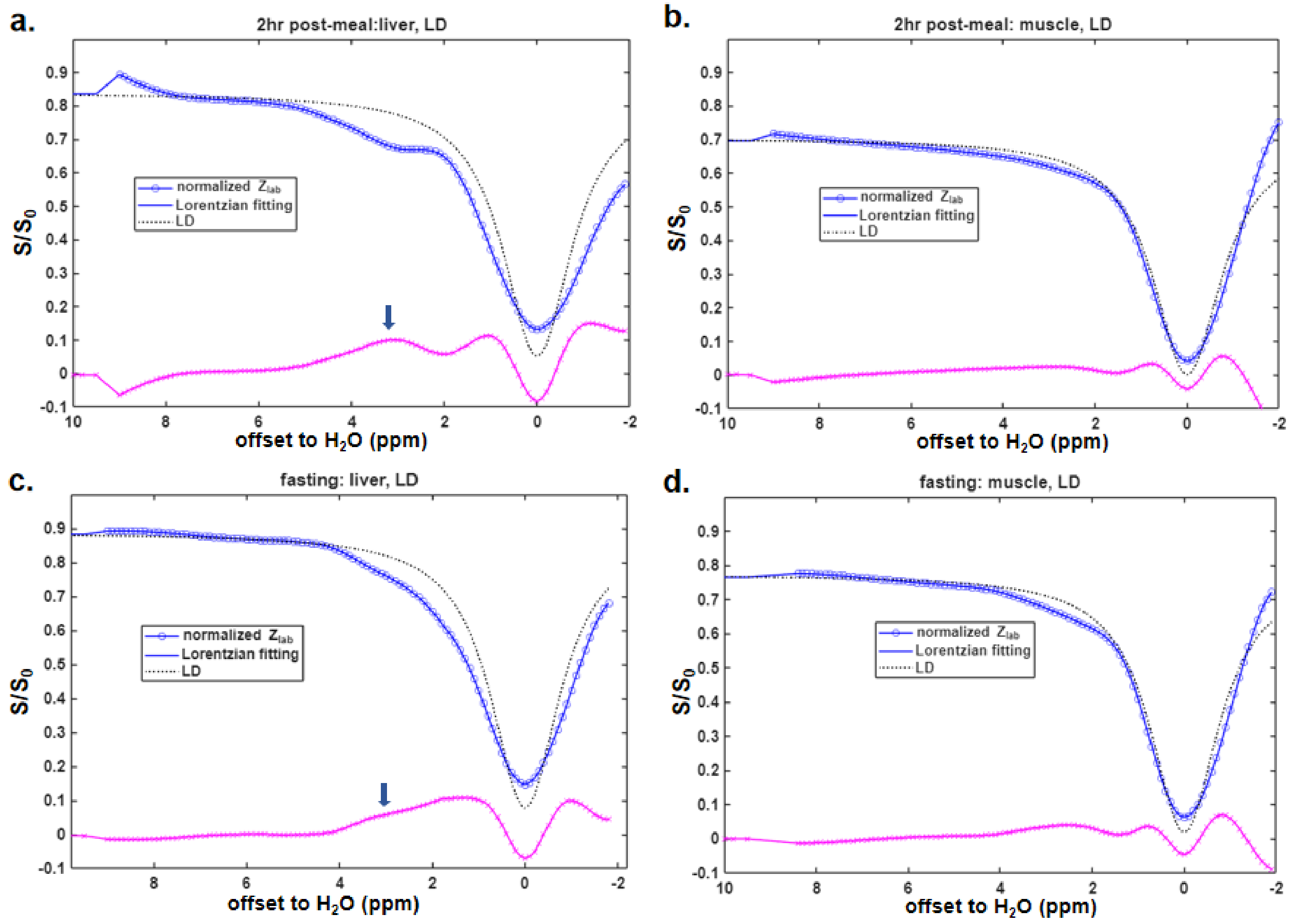

Supplementary Figure 5.2. Lorentzian Difference analysis of Zpos (0.7 μT) for the fasting experiments. Upper Row: 2hr-post meal, Lower Row: fasting, **a,c** for a liver ROI and **b** for a muscle ROI. As seen, compared with liver, muscle has a stronger MTC (>20%), while liver obtains a higher CEST spectra.

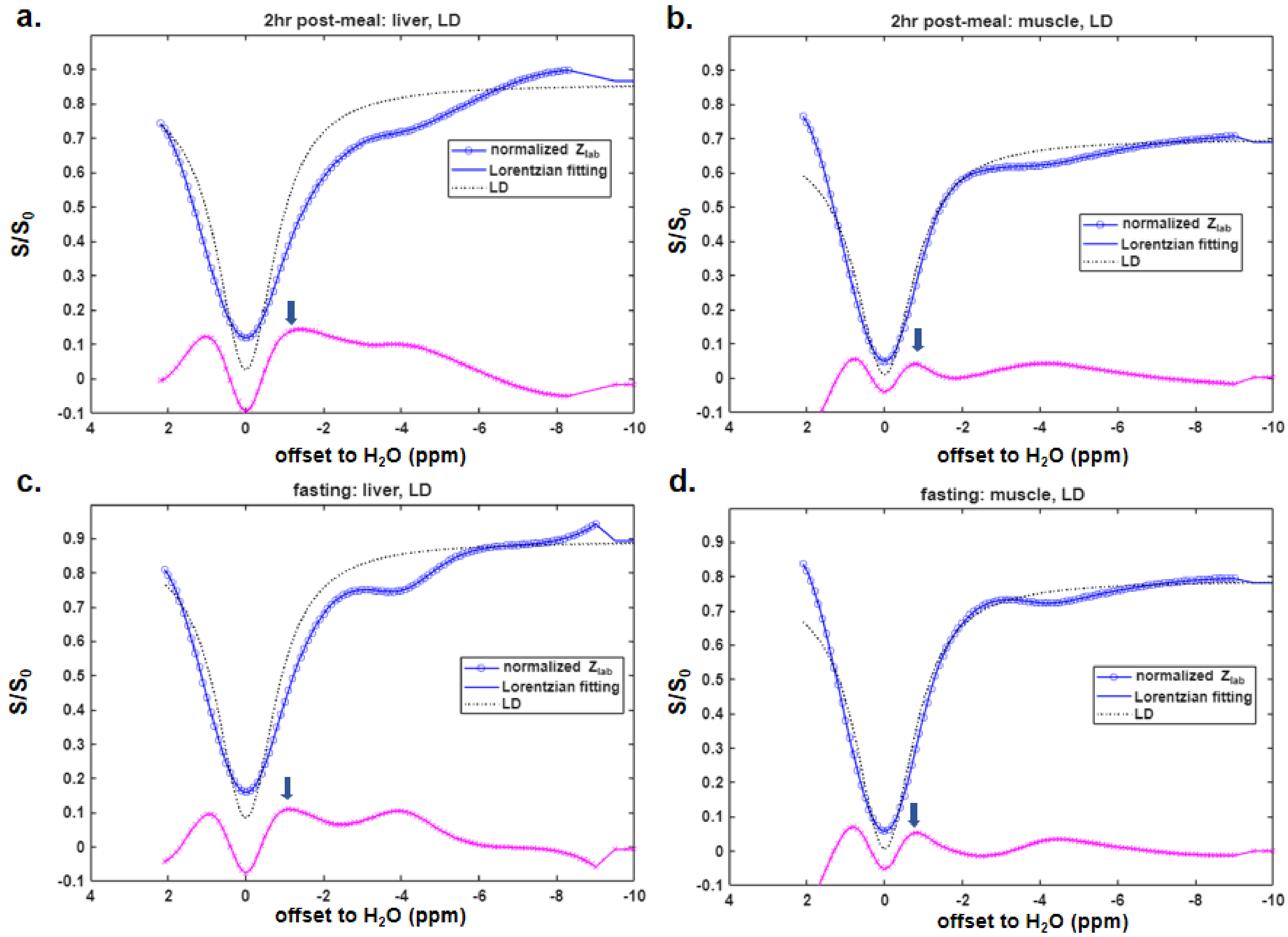

Supplementary Figure 5.3. Lorentzian Difference analysis of Zneg (0.7 µT) for extracting NOE signal in the fasting experiments. Upper Row: 2hr-post meal, Lower Row: fasting, **a,c** for a liver ROI and **b** for a muscle ROI. Glycogen NOE peaks (~**-**1.2 ppm) were observed on all 4 subplots. While liver exhibited dropped glycogen NOE signals after overnight fasting (Also see Fig. 5). Muscle has a stronger MTC than liver has.

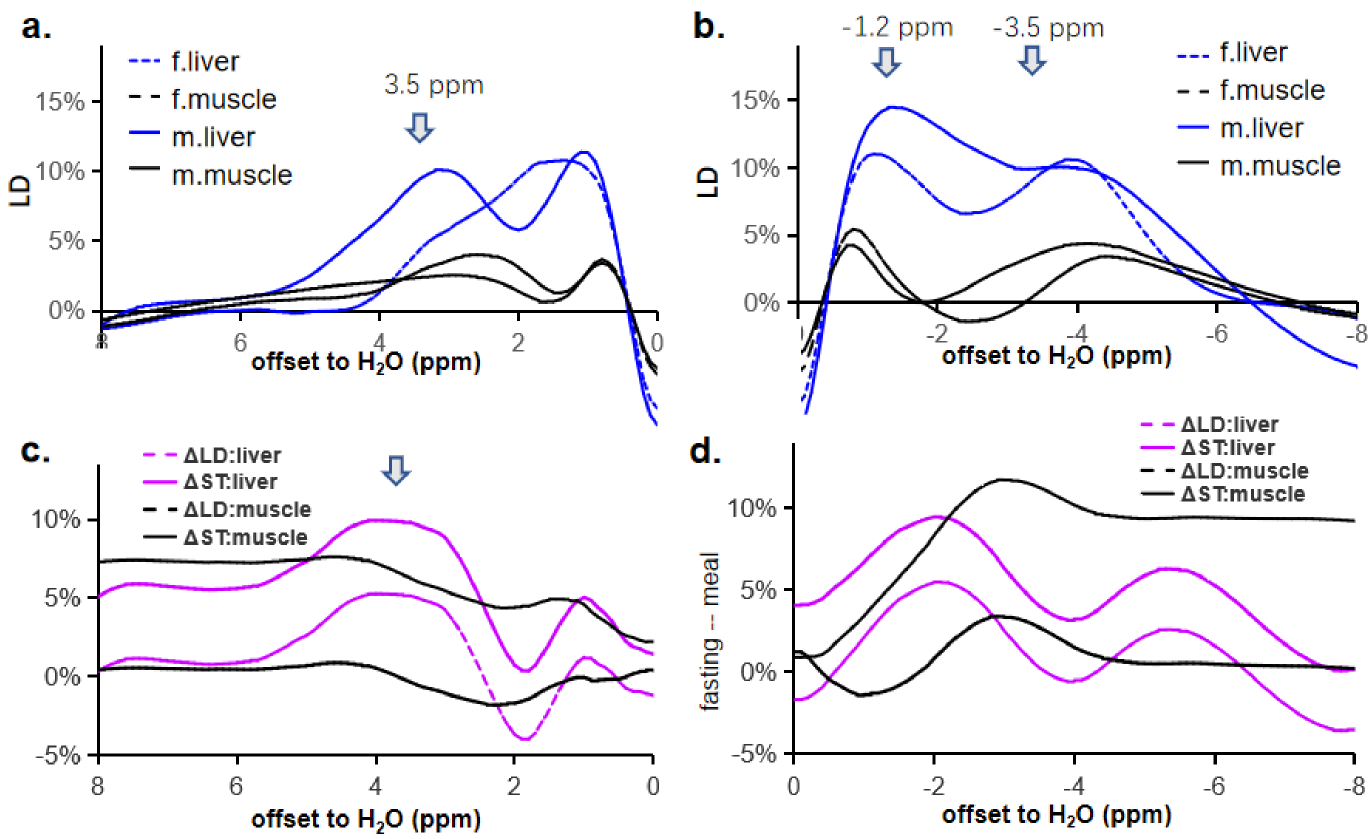

Supplementary Figure 5.4. Spectral comparison between 2hr-post meal and over-night fasting. a,b, LD analysis for fasting (dash) and post-meal (solid), with blue for liver, black for muscle. **c,d,** the spectral difference between fasting and meal (solid: ΔST spectra, dash: LD spectra), with magenta for liver, black for muscle.

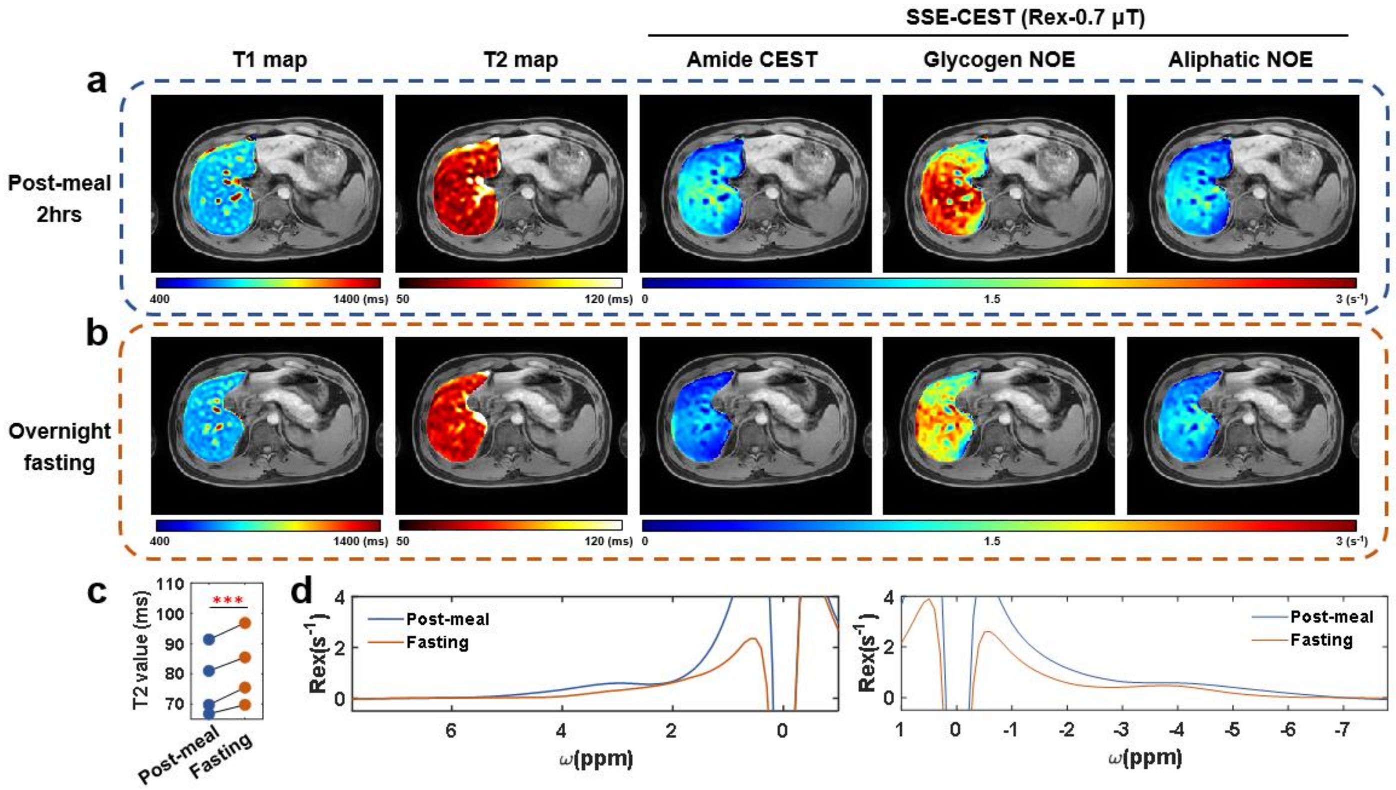

Supplementary Figure 5.5. R1ρ-based quantitative analysis of metabolic and relaxation changes before and after overnight fasting in healthy volunteers. **a**,**b**, Representative maps from one subject showing T_1, T_2, and the exchange-dependent relaxation rate (R_{ex}) for three specific contrasts—amide CEST (3.5 ppm), glycogen NOE (-1.2 ppm) and aliphatic NOE (-3.5 ppm)—acquired **a**, two hours after a meal and **b**, after a 12-hour fasting. R_{ex} values are notably reduced after fasting, while T1 and T2 maps show no appreciable change. **c**, Group-wise comparison of T2 values ($n = 4$ subjects). Although a statistically significant decrease is observed post-meal (two-sided paired t-test, $p < 0.001$), the absolute difference is small (mean ± SD: 77.2 ± 11.2 ms post-meal vs. 81.9 ± 11.9 ms post-fasting), indicating that water relaxation changes, while detectable, do not dominate the observed metabolic contrasts. **d**, R_{ex} spectra of liver parenchyma, demonstrating a consistent reduction in the exchange-related signal after fasting.

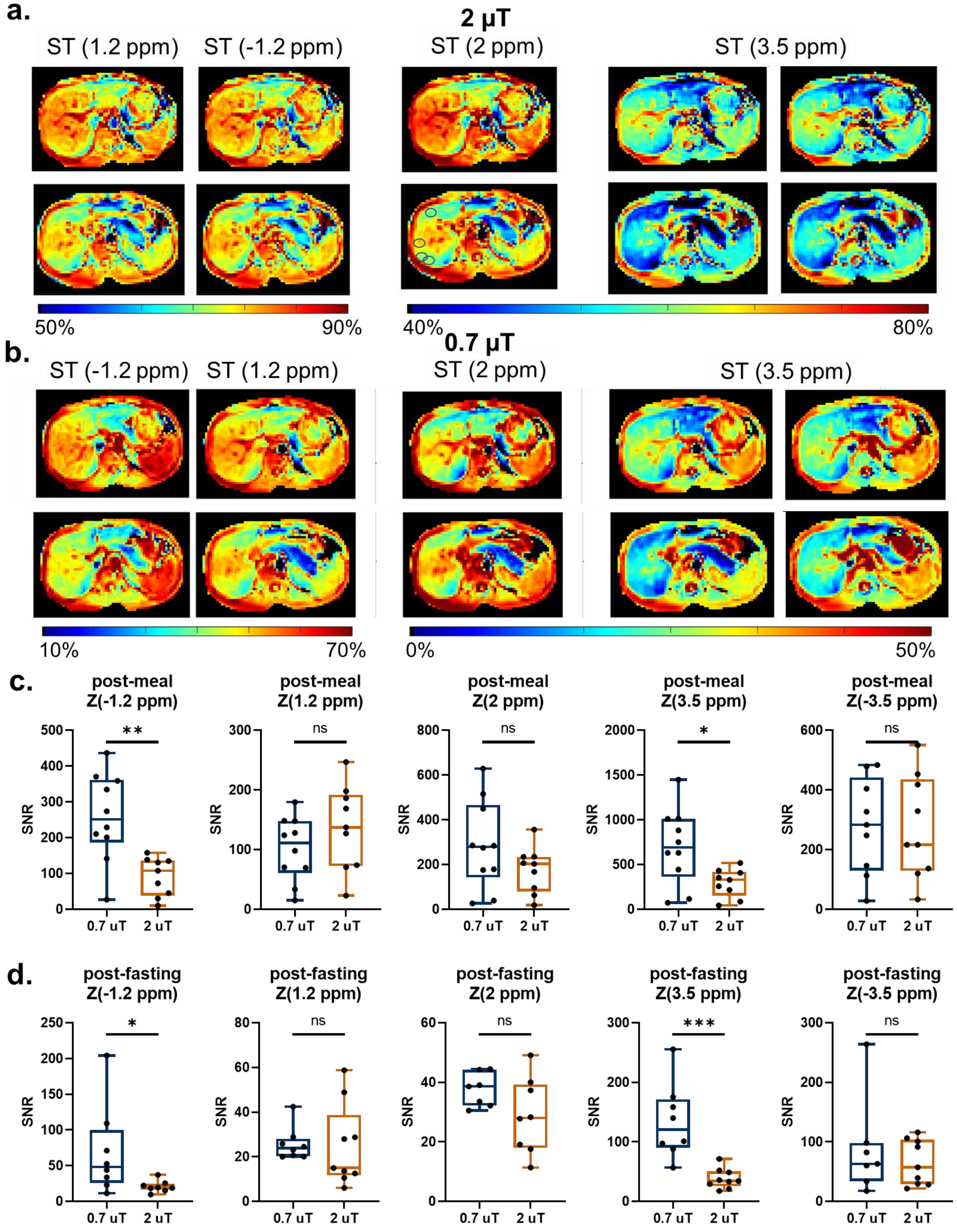

Supplementary Figure 5.6. For a subject in the fasting experiment, comparison of SSE-CEST images and the SNR values acquired using 0.7 μT and those using 2 μT. a, ST images (ST=1-Z) of 0.7 μT acquired 2-hrs post-meal and post overnight fasting, at 5 distinct frequency offsets, corresponding to the labeling of Glycogen NOE (-1.2 ppm), -OH on glycans (1.2 ppm), guanidine amines (2 ppm, also includes -OH leakage under 2 μT), amide (3.5 ppm) and aliphatic NOE (-3.5 ppm). **b,** same layouts as **a**, but using B_1 of 2 μT. **c,** SNR comparison of Z-spectral images acquired using 0.7 μT and 2 μT, at 2 hours post-meal. The SNR values were calculated 10 times, using a randomly-selected noise region (7×7 square) in the background and the signal from the same central liver region (SNR = mean(Signal)/std(Noise), n = 10).**d.** the same layouts

as **c**, but acquired post over-night fasting. For the frequency offsets of **-1.2 ppm** and **3.5 ppm**, the SNR of 0.7 μT are significantly higher than those of 2 μT (unpaired t-test, n=10, *** $p<0.001$, **$p<0.01$, * $p<0.05$).

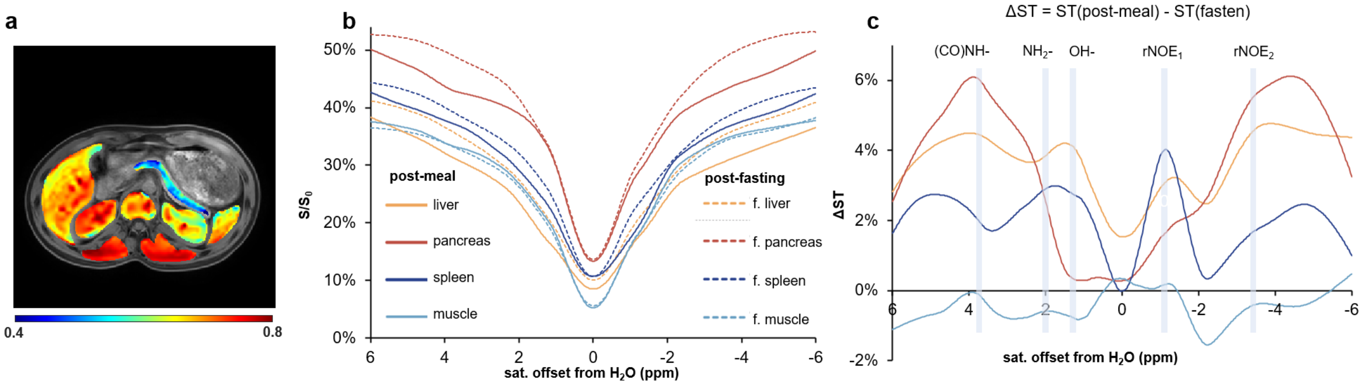

Supplementary Figure 5.7. SSE-CEST imaging of multiple abdominal organs. a, ST image at 3.5 ppm, with liver, kidneys, pancreas, muscle, spinal disk, and spleen sketched. **b**, CEST spectra were plotted for four different organs (liver, pancreas, spleen, and muscle, with ROI indicated in the T1w image in Fig. 5a, solid lines: post meal; dash lines: after overnight fasten; **c**, The subtracted spectra of pre- and post-fasten in Fig. 5 (ΔST), indicating the metabolic characteristics for four different organs. The vertical stripes show the location of feature freq. offsets displayed in Fig. 5a,b.

Part 6: Supplementary materials of OGT experiments

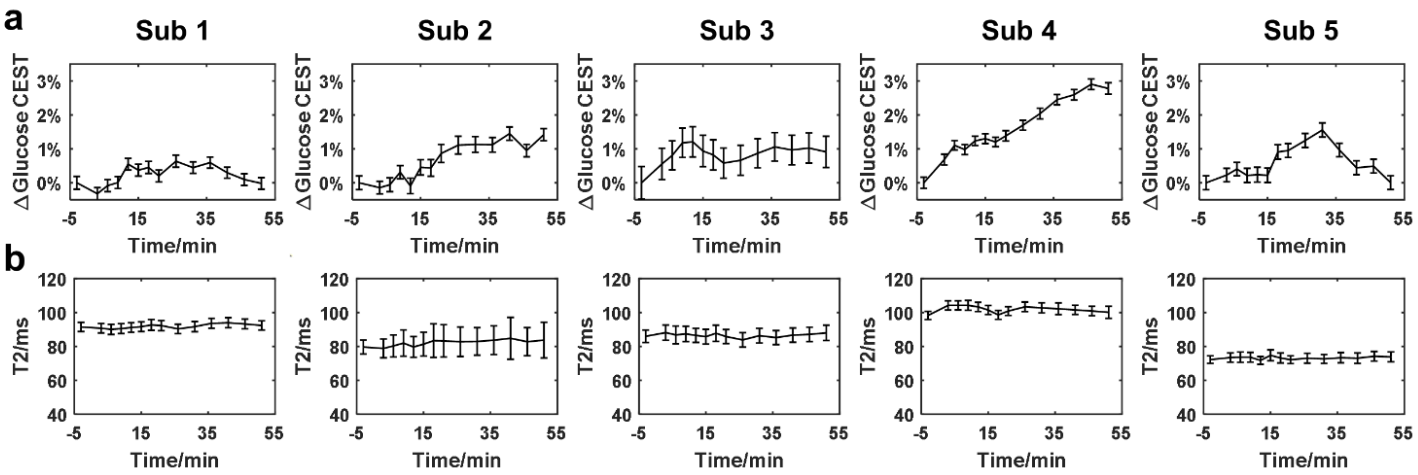

Supplementary Figure 6.1. Dynamic changes in glucose CEST signal and T2 values during oral glucose tolerance (OGT) experiments. Data are shown for all five volunteers. **a**, Change in glucose-weighted CEST contrast, quantified as **MTR asymmetry at 2.1 ppm** relative to the pre-glucose baseline (ΔMTRasym). All subjects demonstrate a clear rising trend following glucose ingestion. **b**, Corresponding T2 values plotted over the same time course. No consistent or significant change in T2 is observed, confirming that the dynamic ΔMTRasym signal reflects specific metabolic changes rather than nonspecific variations in tissue water relaxation. The curves are presented as mean ± std.

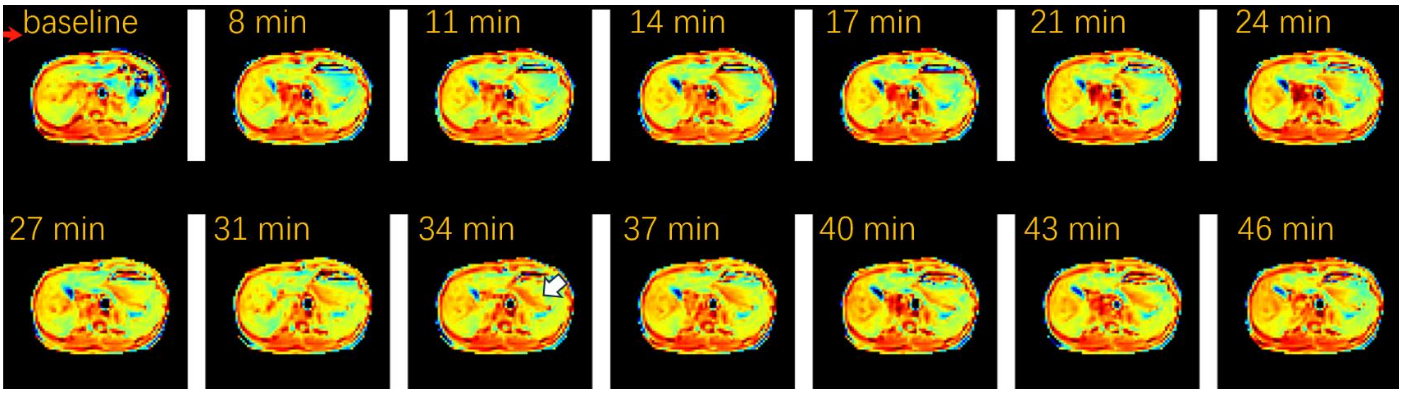

Supplementary Figure 6.2. The ST (2.1ppm) images acquired at all time-points, for the same subject the same slice as in Fig. 6. Images displayed well-alignment, clearly depicting the pancreas (white arrow).

Part 7: Supplementary results of liver patients

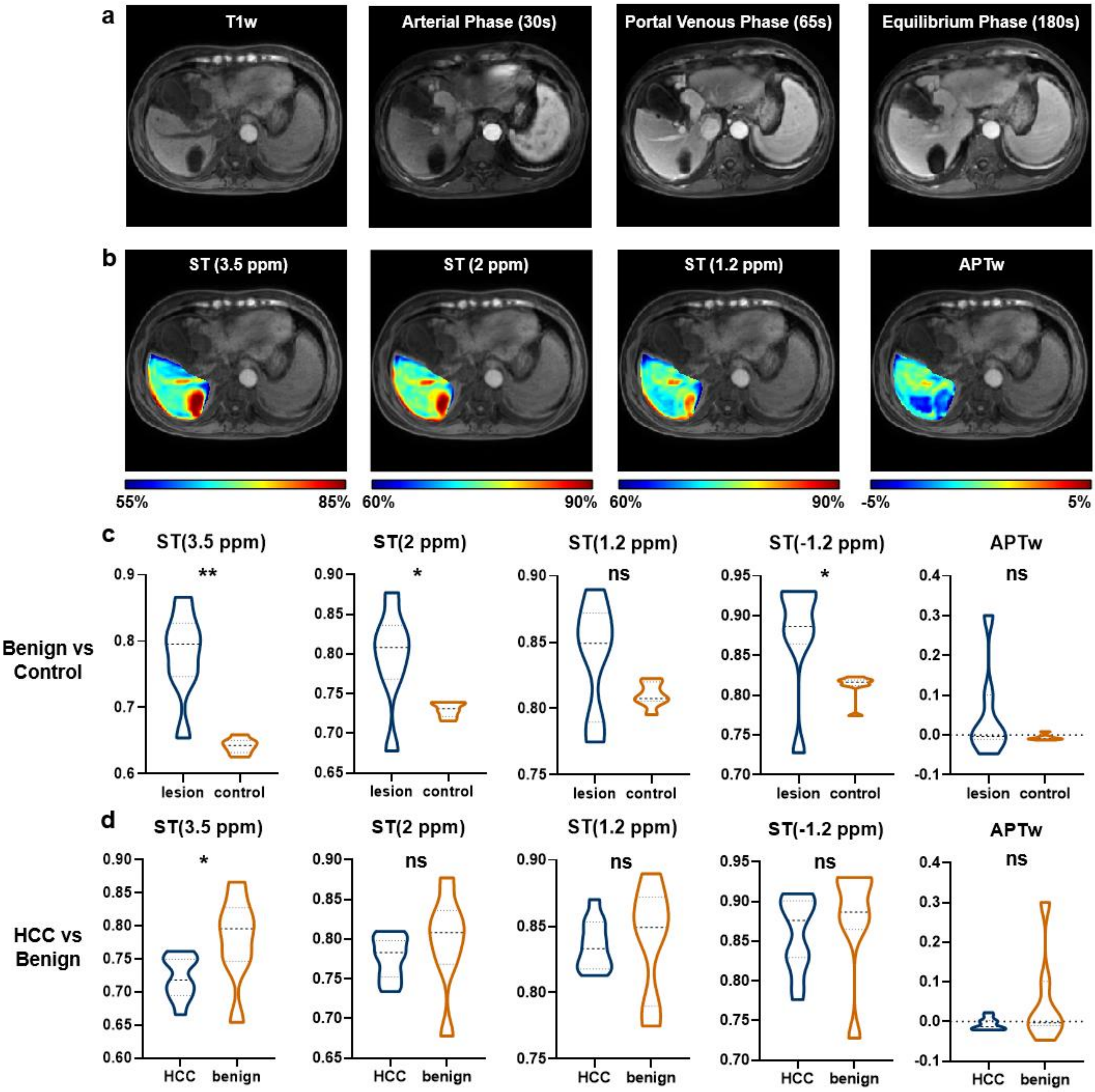

Supplementary Figure 7.1. Results of contrast-enhanced T1w imaging and SSE-CEST imaging from a patient with a liver cyst lesion. a, Contrast-enhanced T1w images, in which the cyst lesion has hypo-intensity. **b**, SSE-CEST imaging results. The cyst lesion has higher ST values and lower APT values compared to healthy-control tissues. **c**, Statistical analysis between benign lesions and their paired control tissues in the same slice (n=9, two-sided paired t-test; *, p<0.05 **, p<0.01). **d**, Statistical analysis between 9 benign lesions and 11 HCC lesions, in which statistical significance can be observed in ST value at 3.5 ppm (two-sided unpaired t-test; *, p<0.05).

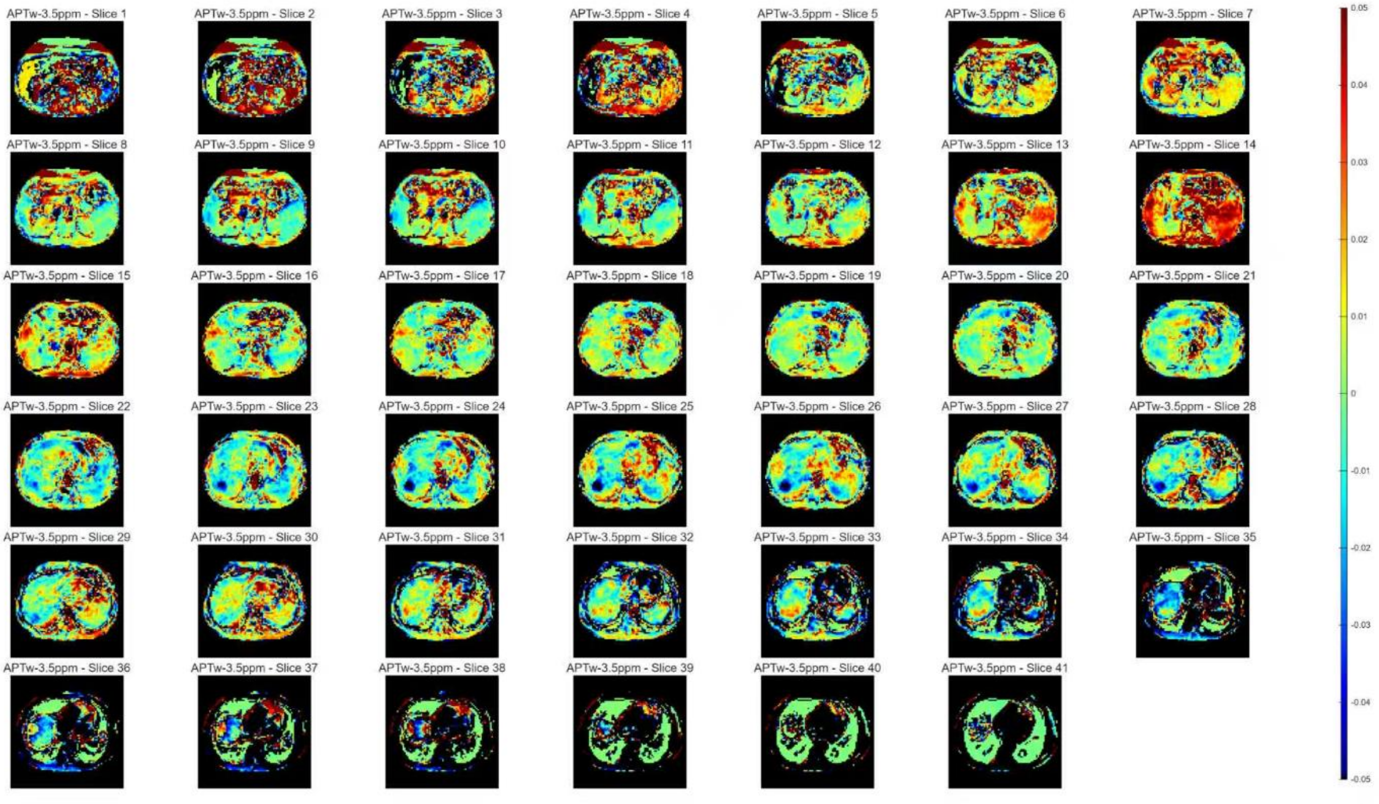

Supplementary Figure 7.2. All 41 slices of APTw images using SSE-CEST 2 μT protocol (The same patient in Fig. 7).

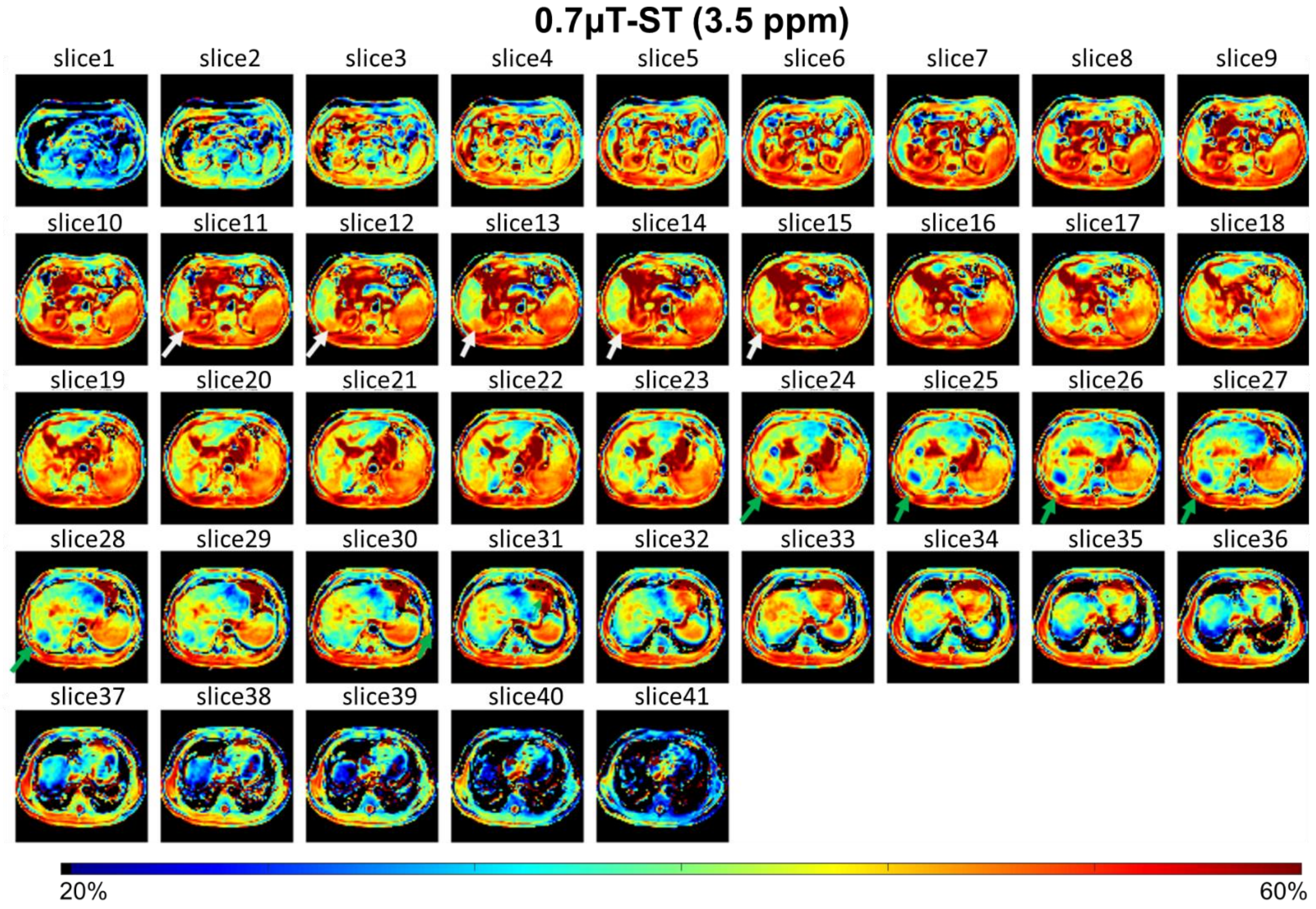

Supplementary Figure 7.3 All 41 slices of ST (3.5ppm) acquired from SSE-CEST (B1 = 0.7 µT) for an HCC patient post-treatment (The same patient as in Fig. 7). The white arrows denote the active lesions exhibited higher amide signals (hyper-intensity in orange color), whereas the green arrows denote the necrotic lesions post-treatment, showing a lower amide signal than normal control tissues (hypo-intense in blue color). The lesions matched well with the DCE images (revised Fig. 7, bottom row).

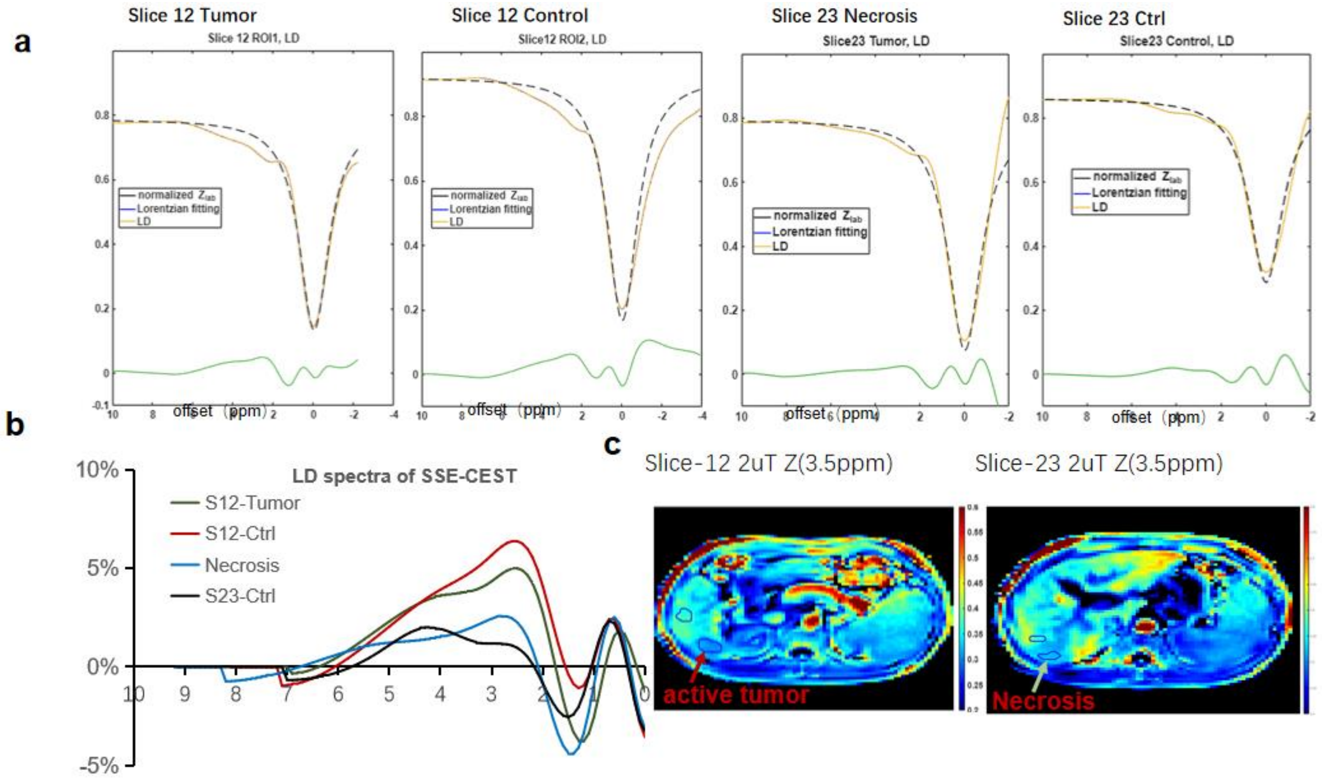

Supplementary Figure 7.4. The represented Z spectra and LD spectra for active lesion region, and for the normal control region (The same patient as in Fig. 7)

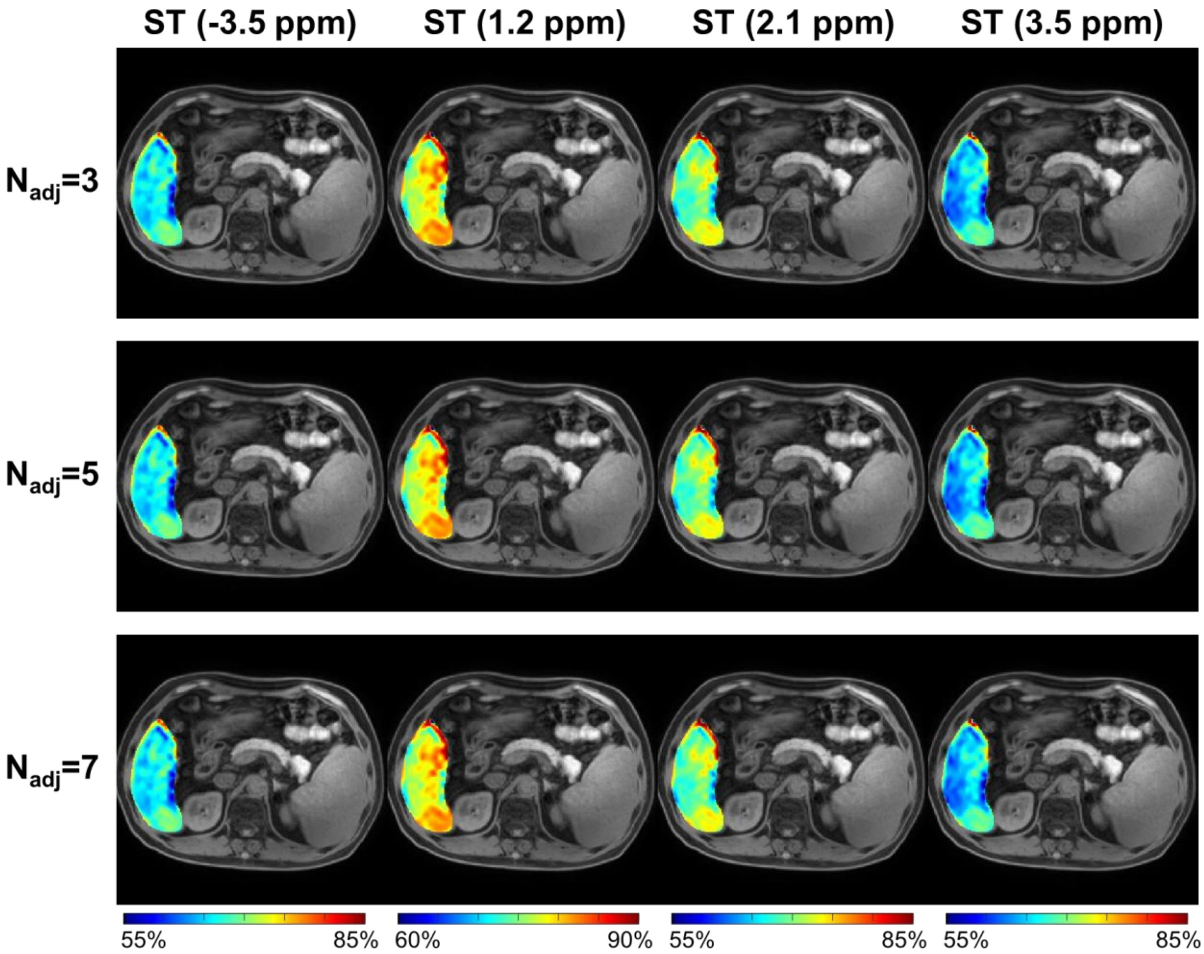

Supplementary Figure 7.5. Comparison of SSE-CEST reconstruction using different number of adjacent slices. (The same patient as in Fig. 7)

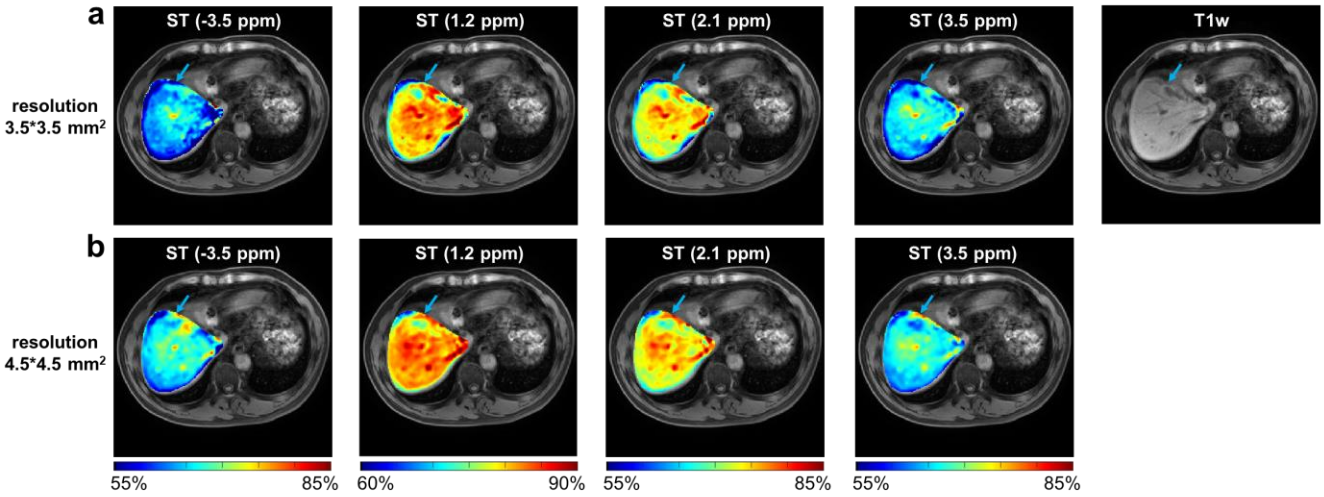

Supplementary Figure 7.6. Demonstration of high-resolution SSE-CEST for depicting small lesions.

Reference：

Bie, C. X., Bo, S. W., Yadav, N. N., van Zijl, P. C. M., Wang, T., Chen, L., Xu, J. D., Zou, C., Zheng, H. R., & Zhou, Y. (2025). Simultaneous monitoring of glycogen, creatine, and phosphocreatine in type II glycogen storage disease using saturation transfer MRI. *Magnetic Resonance in Medicine*, *93*(4), 1782-1792. https://doi.org/10.1002/mrm.30371

Chen, L., Schär, M., Chan, K. W. Y., Huang, J. P., Wei, Z. L., Lu, H. Z., Qin, Q., Weiss, R. G., van Zijl, P. C. M., & Xu, J. D. (2020). In vivo imaging of phosphocreatine with artificial neural networks. *Nature Communications*, *11*(1). https://doi.org/ARTN 107210.1038/s41467-020-14874-0

Chen, Y. B., Dang, X. J., Zhao, B. Q., Chen, Z. S., Zhao, Y. C., Zhao, F. J., Zheng, Z. Z., He, X. W., Peng, J. Y., & Song, X. L. (2023). Frequency importance analysis for chemical exchange saturation transfer magnetic resonance imaging using permuted random forest. *Nmr in Biomedicine*, *36*(6). https://doi.org/ARTN e4744

10.1002/nbm.4744

Deshmane, A., Zaiss, M., Lindig, T., Herz, K., Schuppert, M., Gandhi, C., Bender, B., Ernemann, U., & Scheffler, K. (2019). 3D gradient echo snapshot CEST MRI with low power saturation for human studies at 3T. *Magnetic Resonance in Medicine*, *81*(4), 2412-2423. https://doi.org/10.1002/mrm.27569

Desmond, K. L., Moosvi, F., & Stanisz, G. J. (2014). Mapping of Amide, Amine, and Aliphatic Peaks in the CEST Spectra of Murine Xenografts at 7 T. *Magnetic Resonance in Medicine*, *71*(5), 1841-1853. https://doi.org/10.1002/mrm.24822

Dula, A. N., Arlinghaus, L. R., Dortch, R. D., Dewey, B. E., Whisenant, J. G., Ayers, G. D., Yankeelov, T. E., & Smith, S. A. (2013). Amide proton transfer imaging of the breast at 3 T: Establishing reproducibility and possible feasibility assessing chemotherapy response. *Magnetic Resonance in Medicine*, *70*(1), 216-224. https://doi.org/10.1002/mrm.24450

Jin, T., Wang, P., Zong, X. P., & Kim, S. G. (2012). Magnetic resonance imaging of the Amine-Proton EXchange (APEX) dependent contrast. *Neuroimage*, *59*(2), 1218-1227. https://doi.org/10.1016/j.neuroimage.2011.08.014

Jones, C. K., Polders, D., Hua, J., Zhu, H., Hoogduin, H. J., Zhou, J. Y., Luijten, P., & van Zijl, P. C. M. (2012). In vivo three-dimensional whole-brain pulsed steady-state chemical exchange saturation transfer at 7 T. *Magnetic Resonance in Medicine*, *67*(6), 1579-1589. https://doi.org/10.1002/mrm.23141

Shizhen Chen, M. J., Yaping Yuan,Baolong Wang, Yu Li,Lei Zhang,Zhong-Xing Jiang, Chaohui Ye, Xin Zhou. (2023). Using endogenous glycogen as relaxation agent for imaging liver metabolism by MRI. *Fundamental Research*, *3*(4), 481-487. https://doi.org/https://doi.org/10.1016/j.fmre.2022.10.010

Xu, X., Leforestier, R., Xia, D., Block, K. T., & Feng, L. (2025). MRI of GlycoNOE in the human liver using GraspNOE-Dixon. *Magnetic Resonance in Medicine*, *93*(2), 507-518. https://doi.org/10.1002/mrm.30270

Yadav, N. N., Xu, J. D., Bar-Shir, A., Qin, Q., Chan, K. W. Y., Grgac, K., Li, W. B., McMahon, M. T., & van Zijl, P. C. M. (2014). Natural D-Glucose as a Biodegradable MRI Relaxation Agent. *Magnetic Resonance in Medicine*, *72*(3), 823-828. https://doi.org/10.1002/mrm.25329

Zaiss, M., & Bachert, P. (2013). Chemical exchange saturation transfer (CEST) and MR Z-spectroscopy : a review of theoretical approaches and methods. *Physics in Medicine and Biology*, *58*(22), R221-R269. https://doi.org/10.1088/0031-9155/58/22/R221

Zaiss, M., Schmitt, B., & Bachert, P. (2011). Quantitative separation of CEST effect from magnetization transfer and spillover effects by Lorentzian-line-fit analysis of z-spectra. *Journal of Magnetic Resonance*, *211*(2), 149-155. https://doi.org/10.1016/j.jmr.2011.05.001
