# Supplementary material for: Single-breath-hold 3D abdominal metabolic MRI enables label-free diagnosis of liver cancer
Source: Nat Commun. 2026 Mar 31;17:4661. doi: 10.1038/s41467-026-71124-5 (PMC13201843; doi:10.1038/s41467-026-71124-5)
Supplement: Supplementary file 2 — Reporting Summary [file 41467_2026_71124_MOESM2_ESM.pdf]

nature portfolio

Corresponding author(s): Xiaolei Song

Last updated by author(s): Feb 28, 2026

Reporting Summary

Nature Portfolio wishes to improve the reproducibility of the work that we publish. This form provides structure for consistency and transparency in reporting. For further information on Nature Portfolio policies, see our Editorial Policies and the Editorial Policy Checklist.

Statistics

For all statistical analyses, confirm that the following items are present in the figure legend, table legend, main text, or Methods section.

n/a	Confirmed	
☐	☒	The exact sample size (*n*) for each experimental group/condition, given as a discrete number and unit of measurement
☐	☒	A statement on whether measurements were taken from distinct samples or whether the same sample was measured repeatedly
☐	☒	The statistical test(s) used AND whether they are one- or two-sided<br>*Only common tests should be described solely by name; describe more complex techniques in the Methods section.*
☒	☐	A description of all covariates tested
☒	☐	A description of any assumptions or corrections, such as tests of normality and adjustment for multiple comparisons
☐	☒	A full description of the statistical parameters including central tendency (e.g. means) or other basic estimates (e.g. regression coefficient) AND variation (e.g. standard deviation) or associated estimates of uncertainty (e.g. confidence intervals)
☐	☒	For null hypothesis testing, the test statistic (e.g. *F*, *t*, *r*) with confidence intervals, effect sizes, degrees of freedom and *P* value noted<br>*Give P values as exact values whenever suitable.*
☒	☐	For Bayesian analysis, information on the choice of priors and Markov chain Monte Carlo settings
☒	☐	For hierarchical and complex designs, identification of the appropriate level for tests and full reporting of outcomes
☐	☒	Estimates of effect sizes (e.g. Cohen's *d*, Pearson's *r*), indicating how they were calculated

Our web collection on statistics for biologists contains articles on many of the points above.

Software and code

Policy information about availability of computer code

Data collection	All MRI data were acquired on Philips Ingenia 3.0 T human scanner (Philips Healthcare, Best, The Netherlands).
Data analysis	The acquisition sequences was programmed on Philips PPE platform; The reconstruction and the analysis were performed using custom-written script using MATLAB. (Mathworks, Natick, MA, USA,R2022). Reconstruction and analysis code are avaiable at https://doi.org/10.5281/zenodo.18795840, could be also downloaded from https://github.com/easyCEST<br>Statistical analysis was performed using Prism 8.0.2 (Graphpad Software, San Diego, CA, USA, Version)

For manuscripts utilizing custom algorithms or software that are central to the research but not yet described in published literature, software must be made available to editors and reviewers. We strongly encourage code deposition in a community repository (e.g. GitHub). See the Nature Portfolio guidelines for submitting code & software for further information.

Data

Policy information about availability of data

All manuscripts must include a data availability statement. This statement should provide the following information, where applicable:

- Accession codes, unique identifiers, or web links for publicly available datasets
- A description of any restrictions on data availability
- For clinical datasets or third party data, please ensure that the statement adheres to our policy

The main data supporting the results in this study are available within the paper and its Supplementary Information. The raw data and quantification results from a representative participant in the fasting experiments are available at https://doi.org/10.5281/zenodo.18795840. Source data are provided with this paper.

Research involving human participants, their data, or biological material

Policy information about studies with human participants or human data. See also policy information about sex, gender (identity/presentation), and sexual orientation and race, ethnicity and racism.

Reporting on sex and gender	Participants' sex was determined based on self-report. Sex was not a factor considered in the study design and sex analysis was not carried out due to the small sample size of the healthy subjects or of the patients.
Reporting on race, ethnicity, or other socially relevant groupings	This study did not involve race,ethnicity,or other socially relevant groupings.
Population characteristics	In vivo MRI experiments on healthy volunteers were approved by the Institutional Review Board of Tsinghua University, while patient studies were approved by the Institutional Review Board of Beijing Tsinghua Changgung Hospital. All participants were required written informed consent before MRI scans.<br>For validation of the MRI technique in healthy subjects, 10 subjects were involved in the repeatability test, 5 subjects were involved in fasting experiments and 5 subjects were involved in the Oral-Glucose Tolerance Test (OGTT). In total 21 patients were involved, including 8 with benign lesions, 12 with HCC lesions(11 of them are post treatment), 1 patient (no included in the statistics because of a different protocol for technical validation).
Recruitment	Participants were recruited through printed and electronic advertisements on notice board in XXXXXX. There are not any self-selection bias or other biases.<br>The inclusion criteria for the healthy-volunteer study were as follows:<br>(i) participants aged ≥18 years;<br>(ii) no known disease; (iii) able to undergo MRI (e.g., no metal implants, no claustrophobia).<br><br>The inclusion criteria for the patient study were as follows:<br>(i) participants aged ≥18 years;<br>(ii) histologically or radiologically confirmed liver tumor;<br>(iii) able to undergo MRI. (e.g., no metal implants, no claustrophobia)
Ethics oversight	Healthy volunteer studies were approved by the Institutional Review Board of Tsinghua University (20230011), while patient studies were approved by the Institutional Review Board of Beijing Tsinghua Changgung Hospital (25493-4-01).

Note that full information on the approval of the study protocol must also be provided in the manuscript.

Field-specific reporting

Please select the one below that is the best fit for your research. If you are not sure, read the appropriate sections before making your selection.

☒ Life sciences ☐ Behavioural & social sciences ☐ Ecological, evolutionary & environmental sciences

For a reference copy of the document with all sections, see nature.com/documents/nr-reporting-summary-flat.pdf

Life sciences study design

All studies must disclose on these points even when the disclosure is negative.

Sample size	This pilot study reports invention of a novel MRI technique. Effect sizes were not known a priori, so sample sizes were instead set on the basis of our past experience for technical development. Significant differences were found in the manuscript, implying adequate power. Specifically, for ex vivo procine liver and phantoms, the comparison was carried between the new technique and the conventional ones, whereas the sample sizes are the numbers of ROIs per slice, or the total numbers of ROIs for all 21 acquired slices. In fasting experiments of volunteers, paired comparison was performed between signals acquired at two time-points, with a sample size of total slice numbers (5 subjects,41 slices per subject). For patients,twenty-one patients with focal liver lesions were prospectively enrolled, comprising 8 benign lesions and 12 hepatocellular carcinomas (HCCs). Among the HCC cohort, 11 were post-treatment. One HCC patient was excluded from statistical analysis due to protocol deviation for technical validation.

Data exclusions | No subjects were excluded for analysis.

Replication | The manuscript specifically addresses the repeatability of measurements, in abdominal metabolic MRI.

Randomization | Randomization was not relevant to our study.

Blinding | All MRI data were analyzed in a blinded fashion.

Reporting for specific materials, systems and methods

We require information from authors about some types of materials, experimental systems and methods used in many studies. Here, indicate whether each material, system or method listed is relevant to your study. If you are not sure if a list item applies to your research, read the appropriate section before selecting a response.

Materials & experimental systems

n/a	Involved in the study
☒	☐ Antibodies
☒	☐ Eukaryotic cell lines
☒	☐ Palaeontology and archaeology
☒	☐ Animals and other organisms
☒	☐ Clinical data
☒	☐ Dual use research of concern
☒	☐ Plants

Methods

n/a	Involved in the study
☒	☐ ChIP-seq
☒	☐ Flow cytometry
☒	☐ MRI-based neuroimaging

Plants

Seed stocks | *Report on the source of all seed stocks or other plant material used. If applicable, state the seed stock centre and catalogue number. If plant specimens were collected from the field, describe the collection location, date and sampling procedures.*

Novel plant genotypes | *Describe the methods by which all novel plant genotypes were produced. This includes those generated by transgenic approaches, gene editing, chemical/radiation-based mutagenesis and hybridization. For transgenic lines, describe the transformation method, the number of independent lines analyzed and the generation upon which experiments were performed. For gene-edited lines, describe the editor used, the endogenous sequence targeted for editing, the targeting guide RNA sequence (if applicable) and how the editor was applied.*

Authentication | *Describe any authentication procedures for each seed stock used or novel genotype generated. Describe any experiments used to assess the effect of a mutation and, where applicable, how potential secondary effects (e.g. second site T-DNA insertions, mosiacism, off-target gene editing) were examined.*
